# Supplementary material for: Nance-Horan Syndrome-like 1 protein negatively regulates Scar/WAVE-Arp2/3 activity and inhibits lamellipodia stability and cell migration
Source: Nat Commun. 2021 Sep 28;12:5687. doi: 10.1038/s41467-021-25916-6 (PMC8478917; doi:10.1038/s41467-021-25916-6)
Supplement: Supplementary file 1 — Supplementary Information [file 41467_2021_25916_MOESM1_ESM.pdf]

Nance-Horan Syndrome-like 1 protein negatively regulates Scar/WAVE-Arp2/3 activity and inhibits lamellipodia stability and cell migration

Law et al.

Supplementary Information

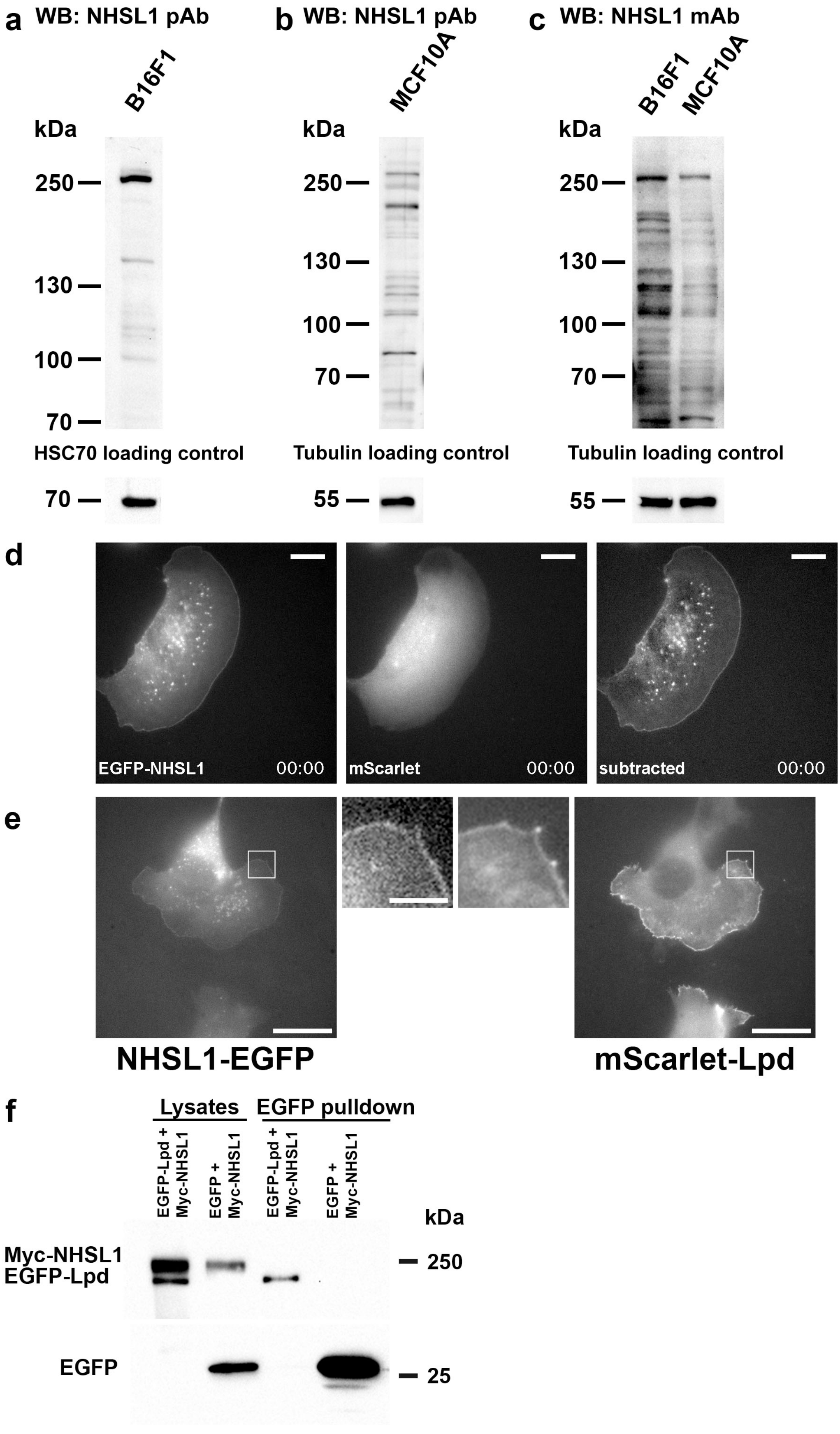

Supplementary Figure 1

Supplementary Figure 1. NHSL1 does not interact with Lpd but co-localises with it at the leading edge of migrating cells.
(a-c) Full Western blots detecting NHSL1 protein in indicated cell lines using the (a,b) polyclonal (4457) or (c) monoclonal antibody (C286F5E1) from Figure 1b-d. Please note that both antibodies should recognise all isoforms, lower molecular weight bands may also represent degradation products. **(d)** Still images from live cell imaging showing EGFP-NHSL1 co-expressed with mScarlet-I only in B16-F1 cells to control for detection of leading edge due to space filling of the fluorescent protein. The subtracted image is generated from the subtraction of the signal of the mScarlet-I from the NHSL1-EGFP signal. Representative images from three independent biological repeats. Scale bar: 20 μm. See also related Suppl. Movie 2. **(e)** Still images from live cell imaging showing NHSL1-EGFP co-expressed with mScarlet-I-tagged Lpd in B16-F1 cells plated on laminin. Representative images from three independent biological repeats. Scale bar: 20 μm. Inset represents a magnified view of the white box. Scale bar in inset: 5 μm. See also related Suppl. Movie 3. **(f)** Western blot showing pulldown using GFP-trap beads from HEK cell lysates co-expressing either EGFP-Lpd or EGFP as control with Myc-NHSL1. Co- immunoprecipitation of Myc-NHSL1 with EGFP-Lpd was examined by western blot with antibody against GFP or Myc. Representative blots from three independent biological repeats. Source data are provided as a Source Data file.

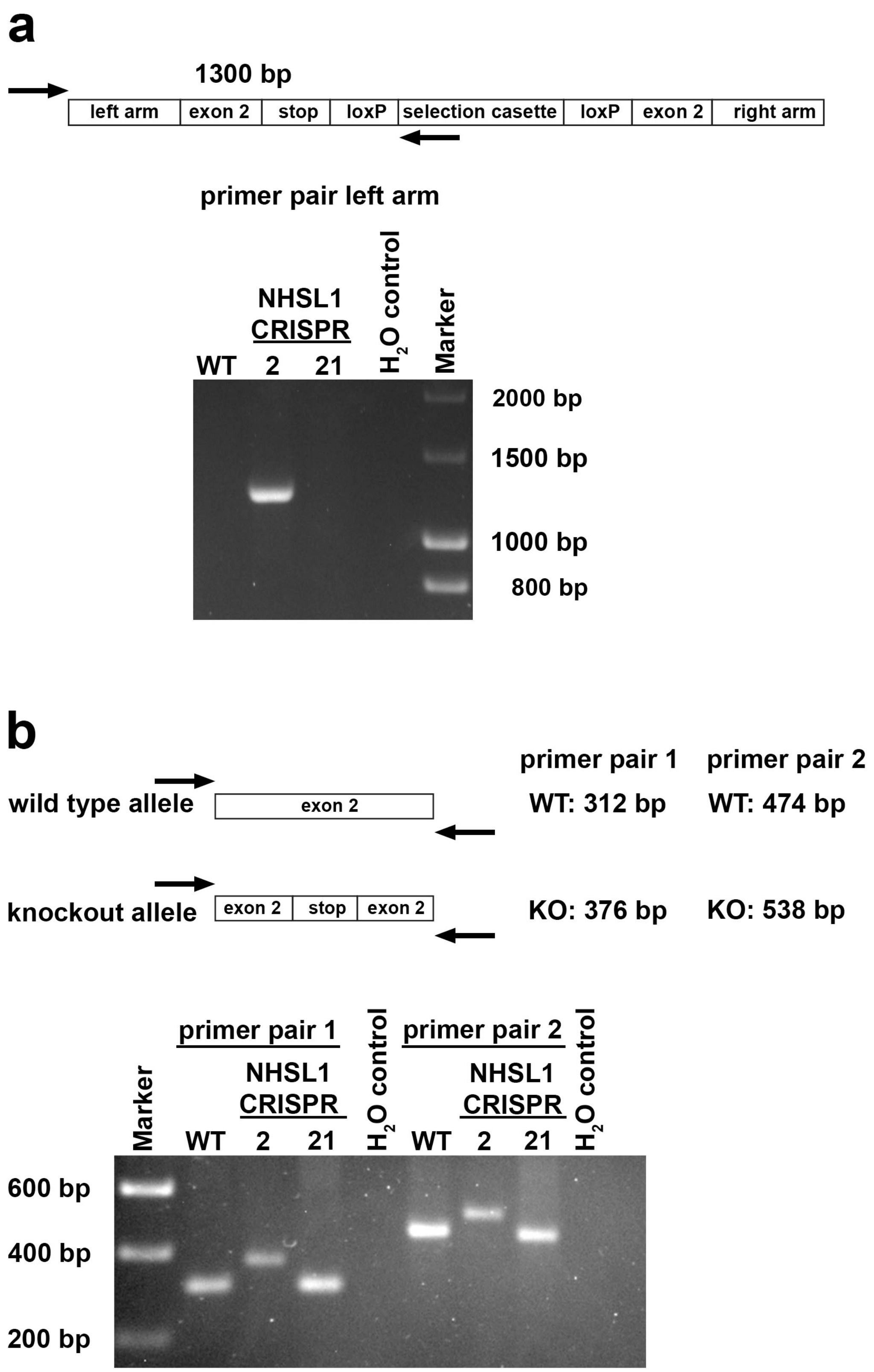

Supplementary Figure 2

Supplementary Figure 2. Genomic analysis of NHSL1 CRISPR knockout clones.
(a,b) Genomic characterization of the NHSL1 CRISPR clones revealed that the targeting cassette was inserted in the correct genomic NHSL1 location for CRISPR 2 but not inserted in CRISPR 21. Upper panel: schematic diagram of the NHSL1 targeting construct to knock in a stop codon into exon 2 and also a LoxP site flanked selection cassette. This diagram also indicates the locations of the genotyping primers for testing proper insertion at the genomic locus and the resulting PCR fragment size of 1300 bp. Please note that the 5' primer sits outside the left arm and the 3' primer is located in the selection cassette and thus this pair only amplifies a fragment if the targeting cassette was inserted in the correct genomic NHSL1 location. Lower panel: Agarose gel showing correct PCR fragment size of 1300 bp only for NHSL1 CRISPR 2 cell line. H_2O instead of genomic DNA served as the negative control for specificity of the amplification. **(b)** After transient Cre recombinase expression in CRISPR 2, all wild type alleles are absent suggesting that the CRISPR 2 line represents a full NHSL1 knockout. Upper panel: schematic diagram of the NHSL1 wild type exon 2 or NHSL1 knockout exon 2 after Cre mediated excision of the selection cassette containing the knocked in stop codon. This diagram also indicates the locations of the genotyping primers for testing wild type or knockout alleles and the resulting PCR fragment size of Primer pair 1: WT 312 bp and KO 376 bp or Primer pair 2: WT 474 bp and KO 538 bp. Lower panel: Agarose gel showing PCR fragment sizes corresponding to WT alleles for WT and CRISPR 21 cells and only KO alleles for CRISPR 2 cells H_2O instead of genomic DNA served as the negative control for specificity of the amplification. **(a,b)** Representative images shown from three independent biological experiments.

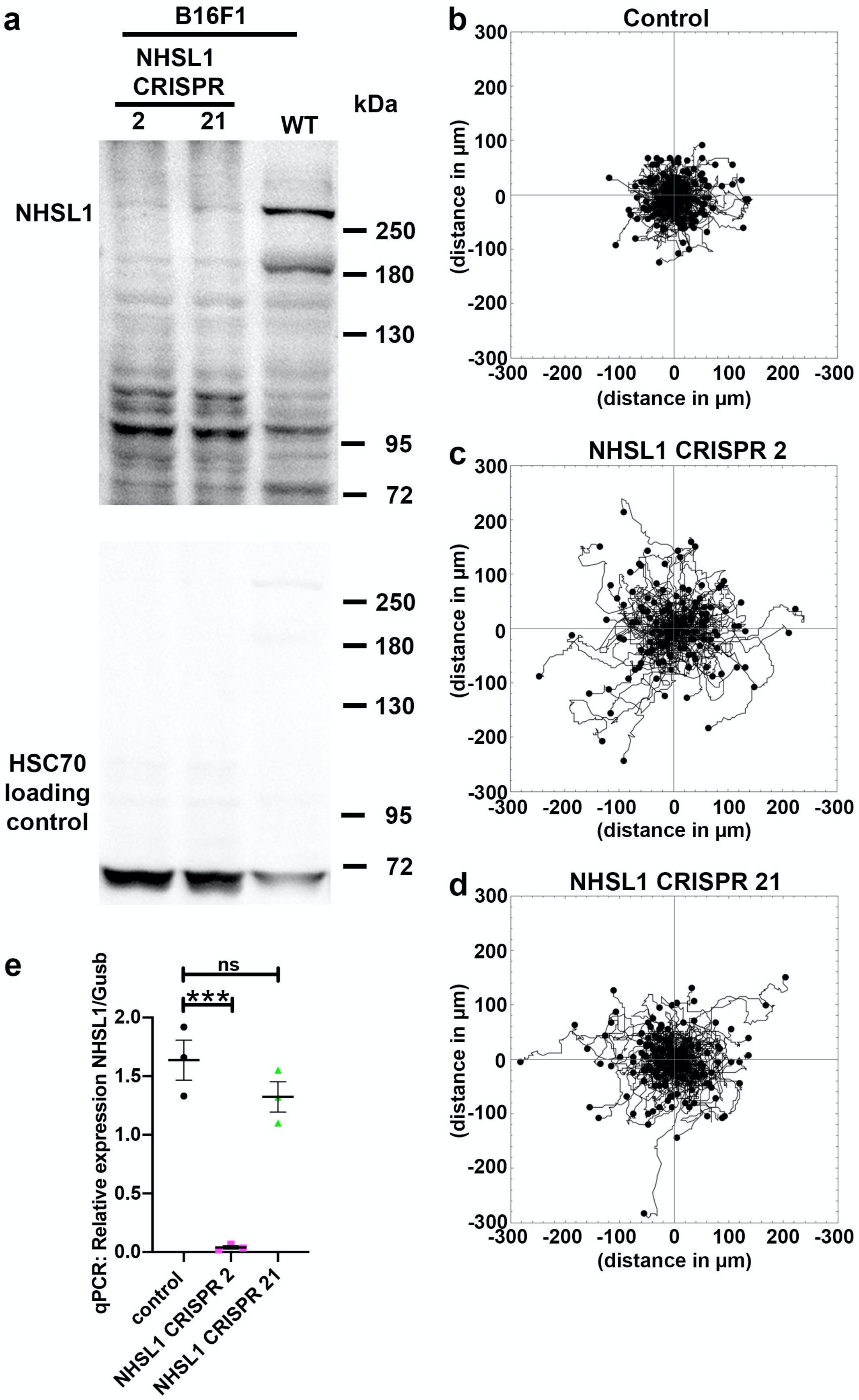

Supplementary Figure 3

Supplementary Figure 3. Characterisation of NHSL1 CRISPR knockout cell lines. **(a)** Full western blot (same as shown in Fig. 2a) showing extent of reduction of NHSL1 expression in the clonal NHSL1 CRISPR B16-F1 cell lines 2 and 21 was probed with polyclonal NHSL1 antibodies and HSC70 antibodies as a loading control. Please note that lower molecular weight bands which may represent degradation products are also reduced in the CRISPR lines. Representative blot from three independent biological repeats. **(b-d)** Migration tracks of both NHSL1 CRISPR clones 1 and 21 in comparison to wild type B16-F1 cells. **(e)** Relative expression of NHSL1 in wild type B16-F1 (control) or NHSL1 CRISPR 2 and 21 cells by qPCR using isoform independent gene specific primer sets compared to expression of housekeeping gene GusB. Results are mean values +/- SEM (error bars) from n=3 independent biological experiments. One-way ANOVA: $p=0.2066$; $F(2,6)=46.6$; Dunnett's multiple comparisons test: *** $p=0.0002$; ns: not significant.

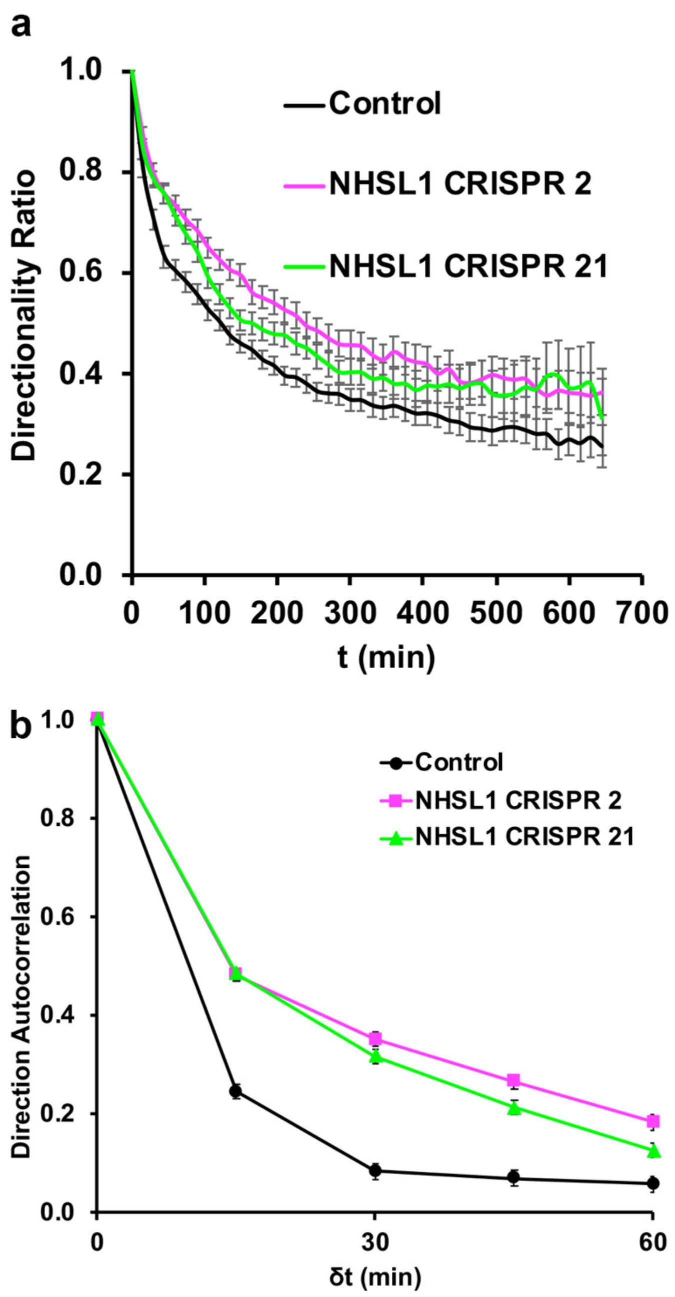

Supplementary Figure 4

Supplementary Figure 4. NHSL1 negatively regulates cell migration persistence. **(a, b)** Cell migration persistence was significantly increased for the NHSL1 CRISPR 2 or 21 cell line, respectively. **(a)** The directionality ratio is plotted to explore how it changes over the time of the movie. **(b)** Direction autocorrelation is shown, a measure of how the angle of displacement vectors correlate with themselves[18] which is independent of speed. (a-b) Results are mean values +/- SEM (error bars) from n= 177 (wild type), 140 (NHSL1 clones 2) and 156 (NHSL1 clones 21) cells from four independent biological repeats. Related to Figure 2 d-f. Source data are provided as a Source Data file.

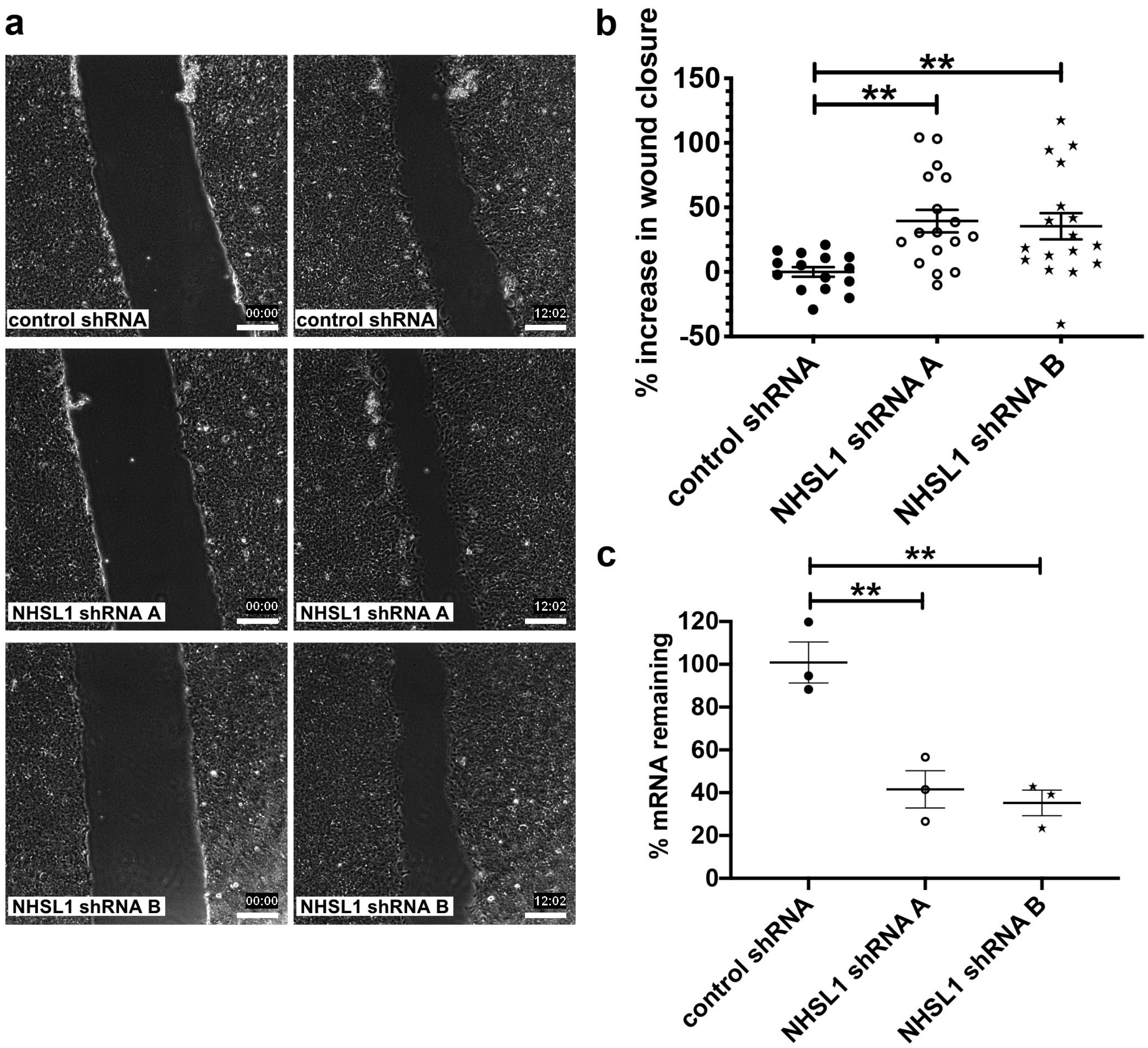

Supplementary Figure 5

Supplementary Figure 5. NHSL1 negatively regulates cell migration in MCF10A cells.

(a) MCF10A normal breast epithelial cells were infected with lentiviruses harbouring control or NHSL1 specific shRNAs (shRNA A or B) and also conferring puromycin resistance and selected by puromycin. Stable MCF10A NHSL1 knockdown cell pools were grown until confluent before being scratched and imaged for 12 hours. Still images from live cell imaging showing one still image at the beginning (0 hours, left panel) and at the end of imaging (12 hours, right panel.) Scale bar: 300 μm. Representative images shown from four independent biological experiments. **(b)** The area of the scratch was measured at 0 and 12 h. Area closure is shown as percentage increase over control cells. Results are mean ± SEM (error bars), from four independent biological repeats; full circles: control shRNA; empty circles: NHSL1 shRNA A; stars: NHSL1 shRNA B; One-way ANOVA: $p=0.0031$, $F(2,46)=6.573$; and Dunnett's multiple comparisons test: control vs. shRNA A: **, $p=0.0033$. control vs. shRNA B: **, $p=0.0085$. See Suppl. Movie 4. **(c)** NHSL1 mRNA levels in stable MCF10A cell lines from (a,b) were measured by quantitative PCR. NHSL1 knockdown reduced mRNA levels to (41.6±8.7)% SEM (shRNA A), or (35.2±6.0)% SEM (shRNA B) compared to scrambled control shRNA. Results are mean ± SEM (error bars); full circles: control shRNA; empty circles: NHSL1 shRNA A; stars: NHSL1 shRNA B; One-way ANOVA: $p=0.0024$, $F(2,6)=19.37$; and Dunnett's multiple comparisons test: control versus shRNA A: **, $p=0.0040$; control versus shRNA B: **, $p=0.0024$. N=3 independent biological experiments. Source data are provided as a Source Data file.

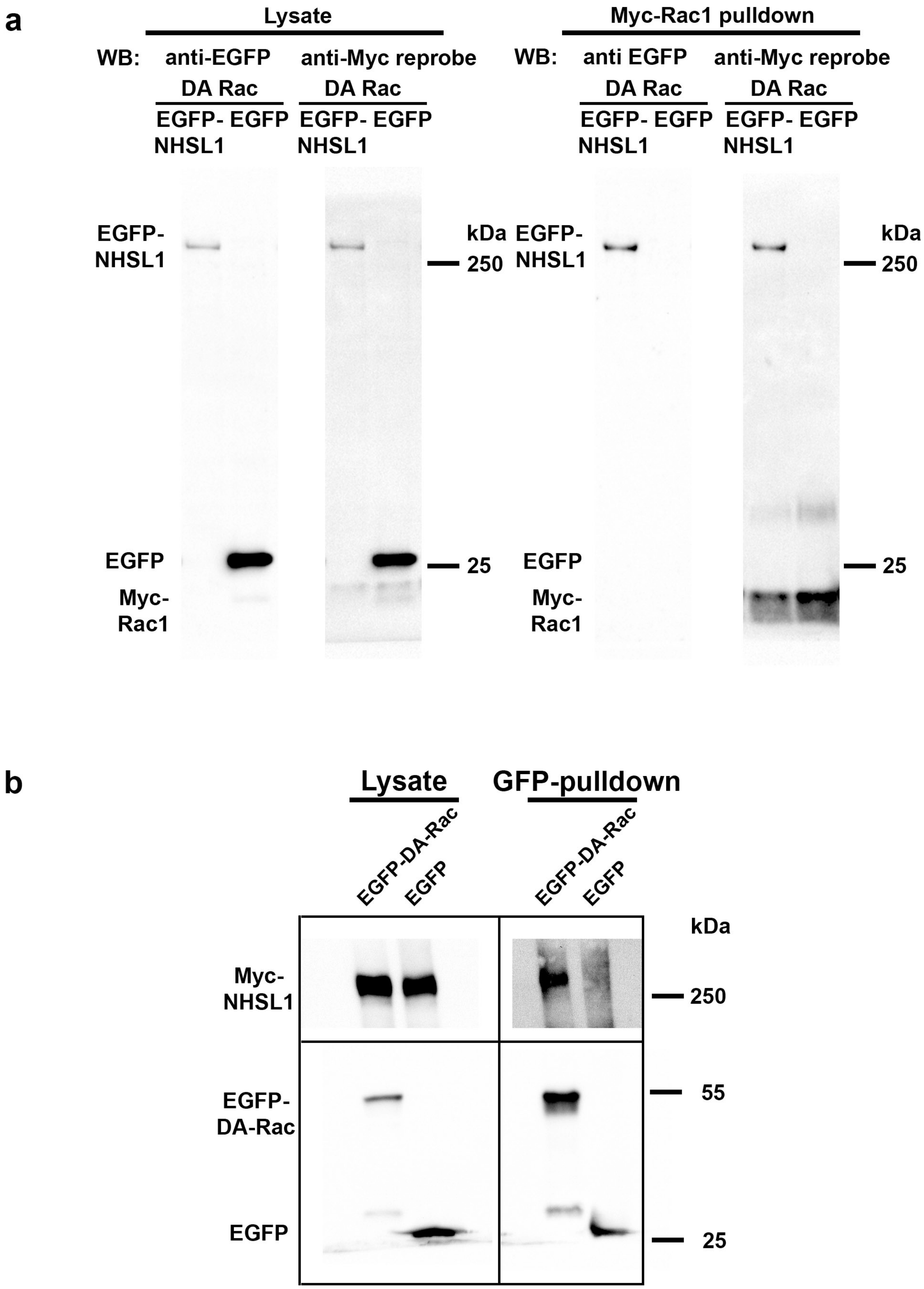

Supplementary Figure 6

Supplementary Figure 6. NHSL1 is a binding partner of active Rac.
(a) Full western blots are shown for Fig. 3g: Western blot showing that dominant active (DA) Rac pulls down NHSL1 using Myc-trap beads from HEK cell lysates expressing Myc-tagged DA Rac1 co-expressed with EGFP-tagged NHSL1 or EGFP only as control. Representative blots from three independent biological repeats. **(b)** Western blot showing that dominant active (DA) Rac pulls down NHSL1 using GFP-trap beads from HEK cell lysates expressing Myc-tagged NHSL1 co-expressed with EGFP-tagged dominant active (DA) Rac1 or EGFP only as control. Representative blots shown from three independent biological experiments.

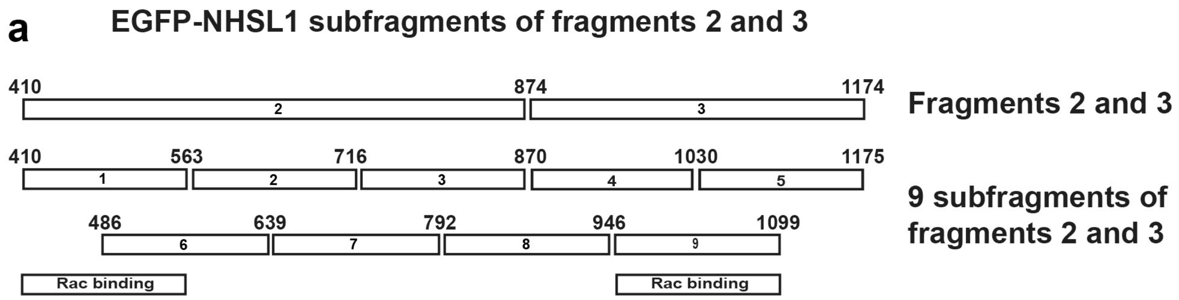

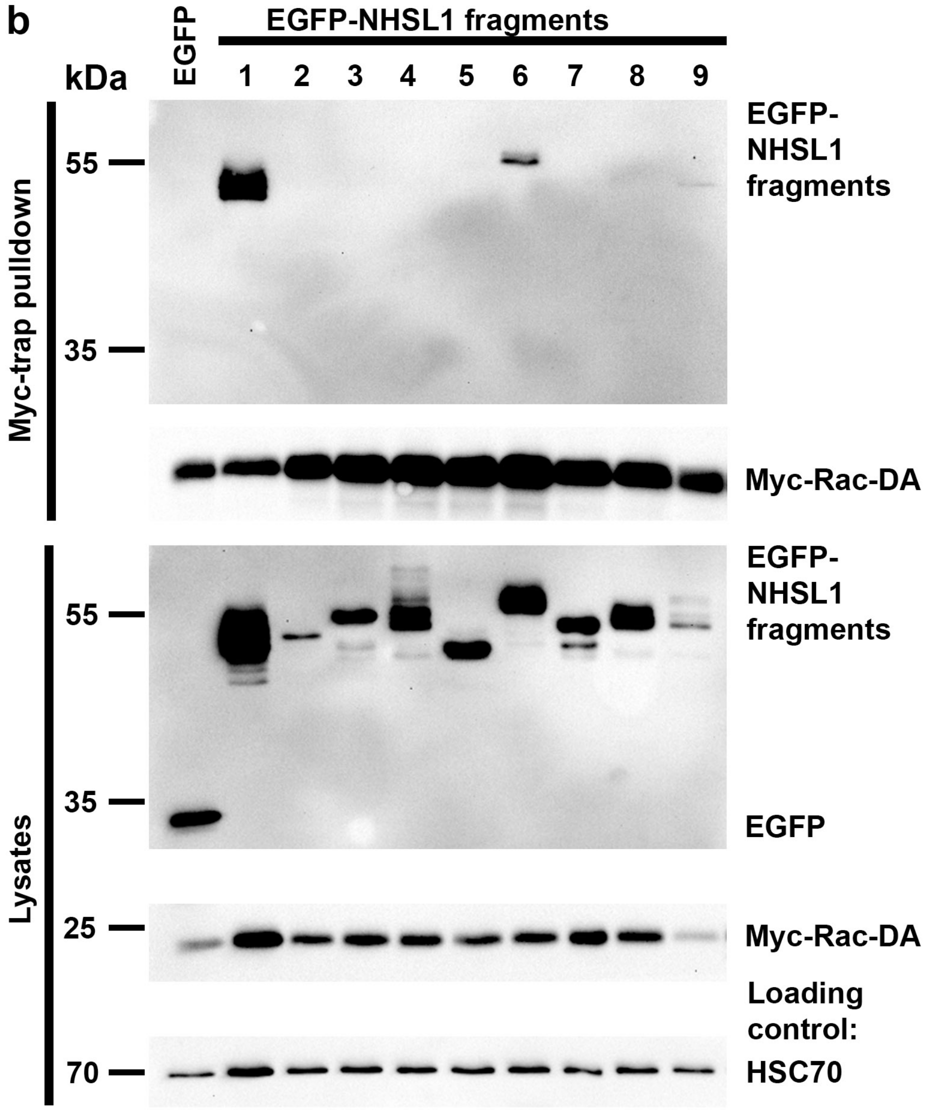

Supplementary Figure 7

Supplementary Figure 7. Active Rac binds to two sites in NHSL.
(a-b) Nine overlapping EGFP-tagged subfragments covering fragments 2 and 3 of NHSL1 were generated (a) and expressed in HEK cells along with Myc-tagged DA Rac. After Myc- trap pulldown of Myc-DA-Rac, co-precipitation of EGFP-NHSL1 subfragments 1-9 was detected in a western blot with Myc antibody (b). Representative blots from three independent biological repeats. Source data are provided as a Source Data file.

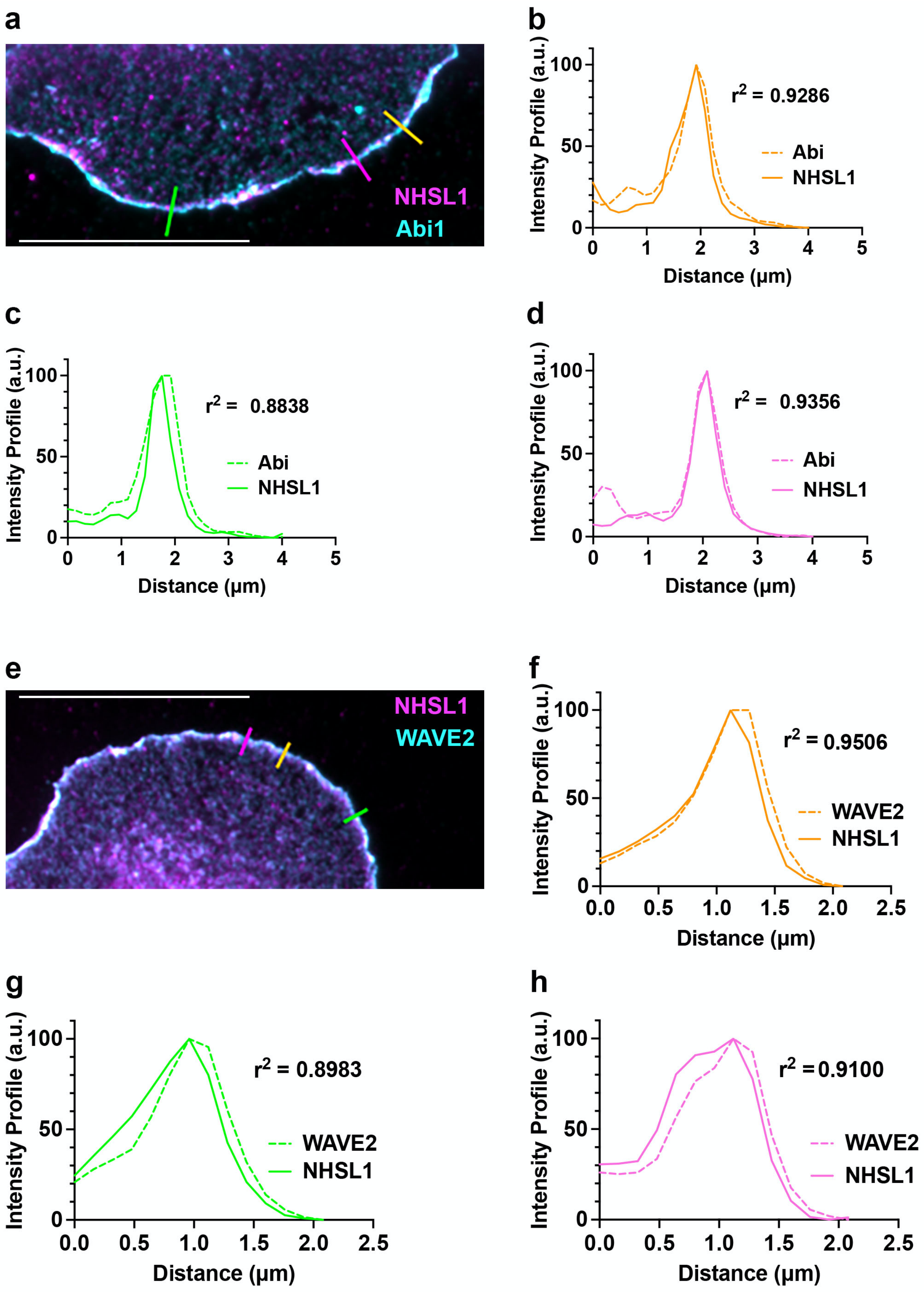

Supplementary Figure 8

Supplementary Figure 8. NHSL1 co-localises with Abi and the Scar/WAVE complex. (a-d) Endogenous NHSL1 (NHSL1 pAb, magenta) co-localises with Abi1 (cyan) at the very edge of lamellipodia in B16-F1 mouse melanoma cells. **(e-h)** Endogenous NHSL1 (NHSL1 mAb, magenta**)** co-localises with Scar/WAVE2 (cyan) and at the very edge of lamellipodia in B16-F1 mouse melanoma cells. (a, e) Dual color merge of the insets shown in Fig. 4 (c,e): Scale bar in (a,e): 20 μm. (b-d;f-h) Three line scans (green, magenta, orange) were placed in arbitrary locations perpendicular to the leading edge and the intensity profile plotted; interrupted line: Abi1/WAVE2; continuous line: NHSL1; The Pearson's correlation coefficient (r^2) indicating degree of correlation of co-localisation was calculated and is displayed next to each pair of lines. **(a,e)** Representative images shown from three independent biological experiments.

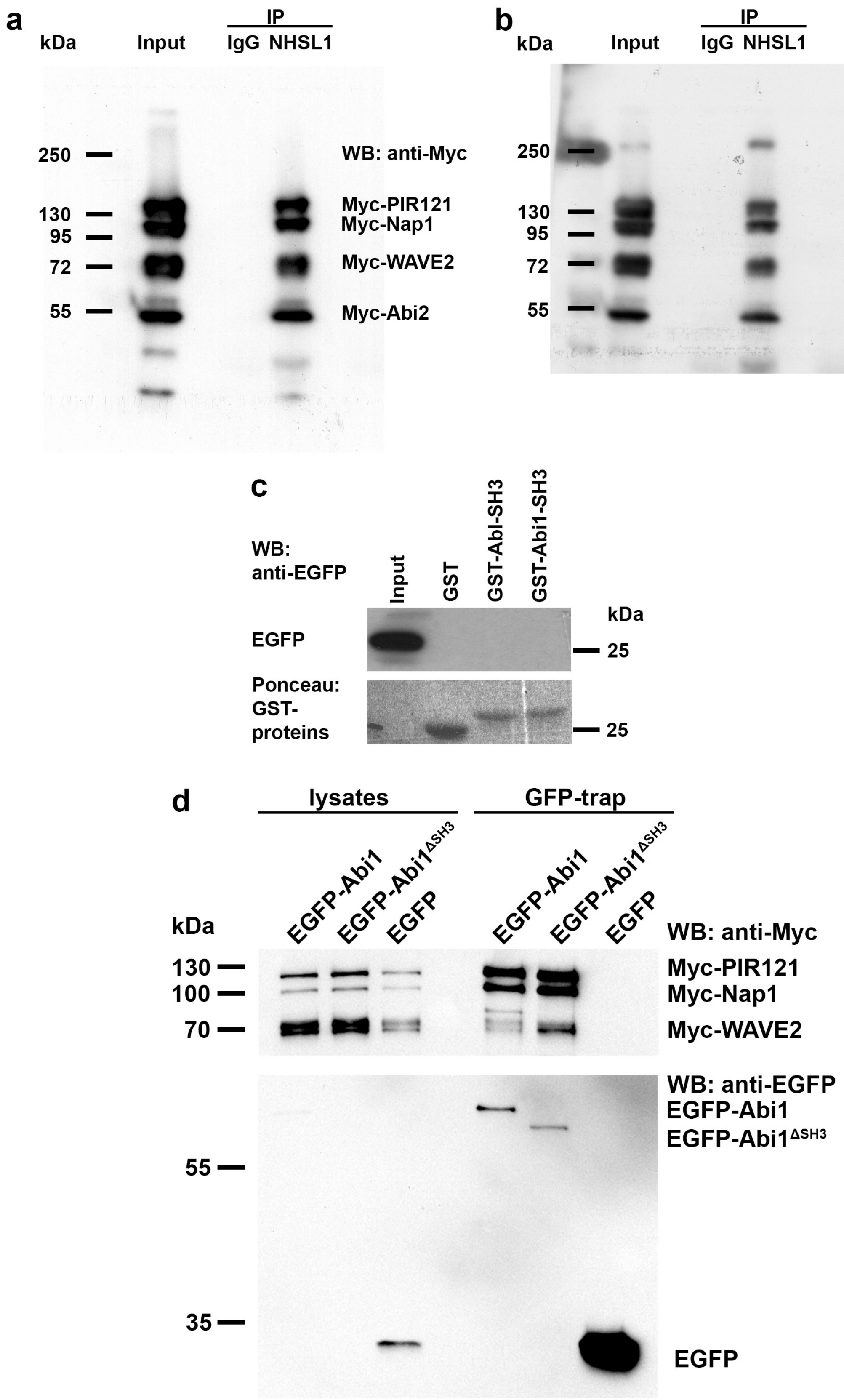

Supplementary Figure 9

Supplementary Figure 9. Exogenous expression of all tagged components of the Scar/WAVE complex allows Scar/WAVE complex formation.
(a,b) Full western blots of experiment shown in Fig. 5a: The Scar/WAVE complex co-immunoprecipitates with NHSL1. HEK cells were transfected with EGFP-NHSL1, and Myc-Pir121, -Nap-1, -WAVE2, -Abi2. NHSL-1 was immunoprecipitated (pAb 4457) from lysates and co-immunoprecipitation tested on a western blot with (a) Myc and reprobed with (b) EGFP antibodies. Representative blots from three independent experiments. **(c)** Negative control for experiment in Fig. 5d: GST-pull downs using purified Glutathione-sepharose coupled GST- fusion proteins of Abi1 and c-Abl SH3 domains or GST alone from HEK cell lysates that were transfected with EGFP. Following GST-pulldown EGFP was detected in a western blot with anti-EGFP antibodies. Ponceau staining of membrane reveals GST or GST-tagged Abi- or Abl-SH3 domains used. Representative blots from three independent experiments. **(d)** HEK cells were transfected with EGFP-tagged Abi1 or Abi1-delta-SH3 or EGFP only as negative control and the remaining Myc-tagged Scar/WAVE components, PIR121, Nap1, Scar/WAVE2, HSPC300. After GFP-trap pulldown, co-precipitation was detected in a western blot with Myc antibody. Representative blots from three independent biological repeats. Source data are provided as a Source Data file.

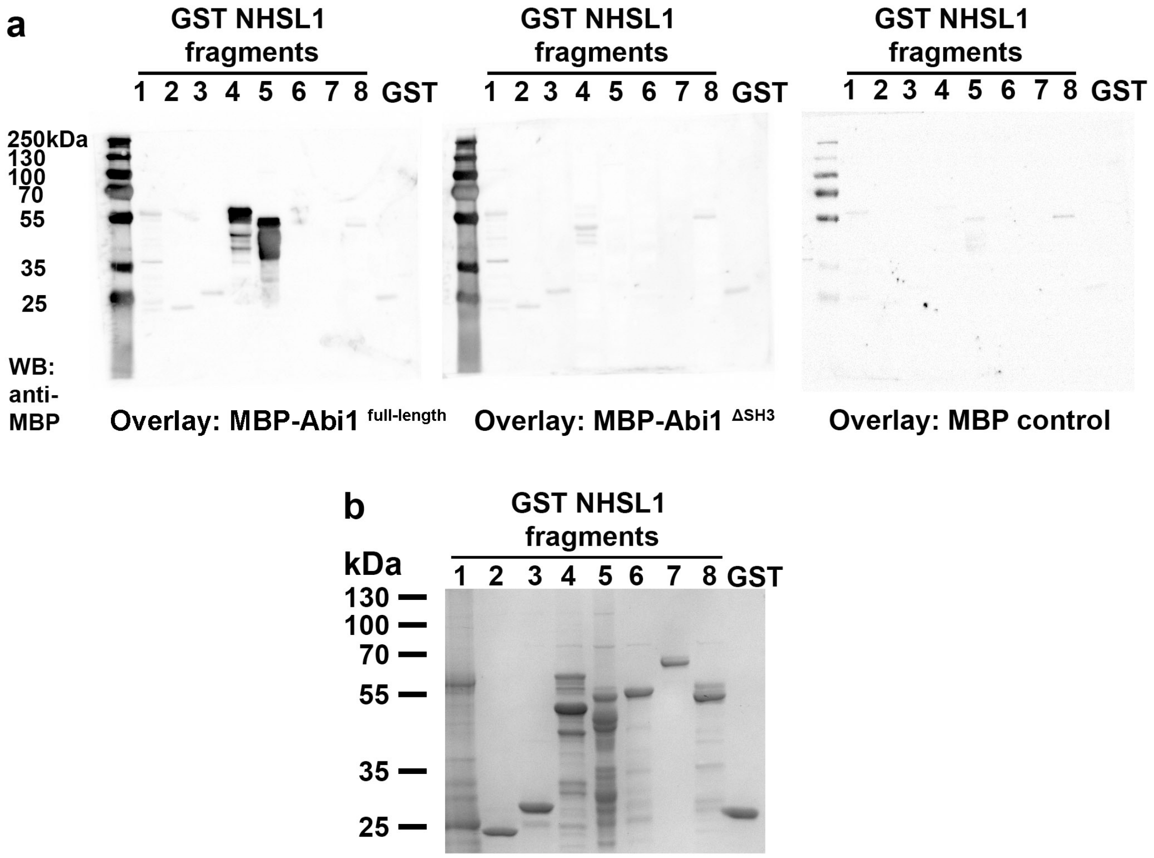

Supplementary Figure 10

Supplementary Figure 10. Abi SH3 domain binds to two fragments of NHSL1.
(a) Far western overlay with purified MBP-tagged full-length Abi1 (MBP-Abi1 full length) or an MBP fusion protein with Abi1 in which the SH3 domain had been deleted (MBP-Abi1-delta-SH3) and MBP as control on a blot of different purified GST-NHSL1 fusion proteins covering the entire length of NHSL1. Representative blots from three independent experiments. Fragments 4 and 5 contain three putative SH3 binding sites. **(b)** Coomassie gel showing GST fragments covering the entire length of the NHSL1 amino acid sequence (see Fig. 1e for fragment sizes and location within NHSL1) and GST only as control which are used in the Far Western Blot in (a). Representative gel shown from three independent experiments.

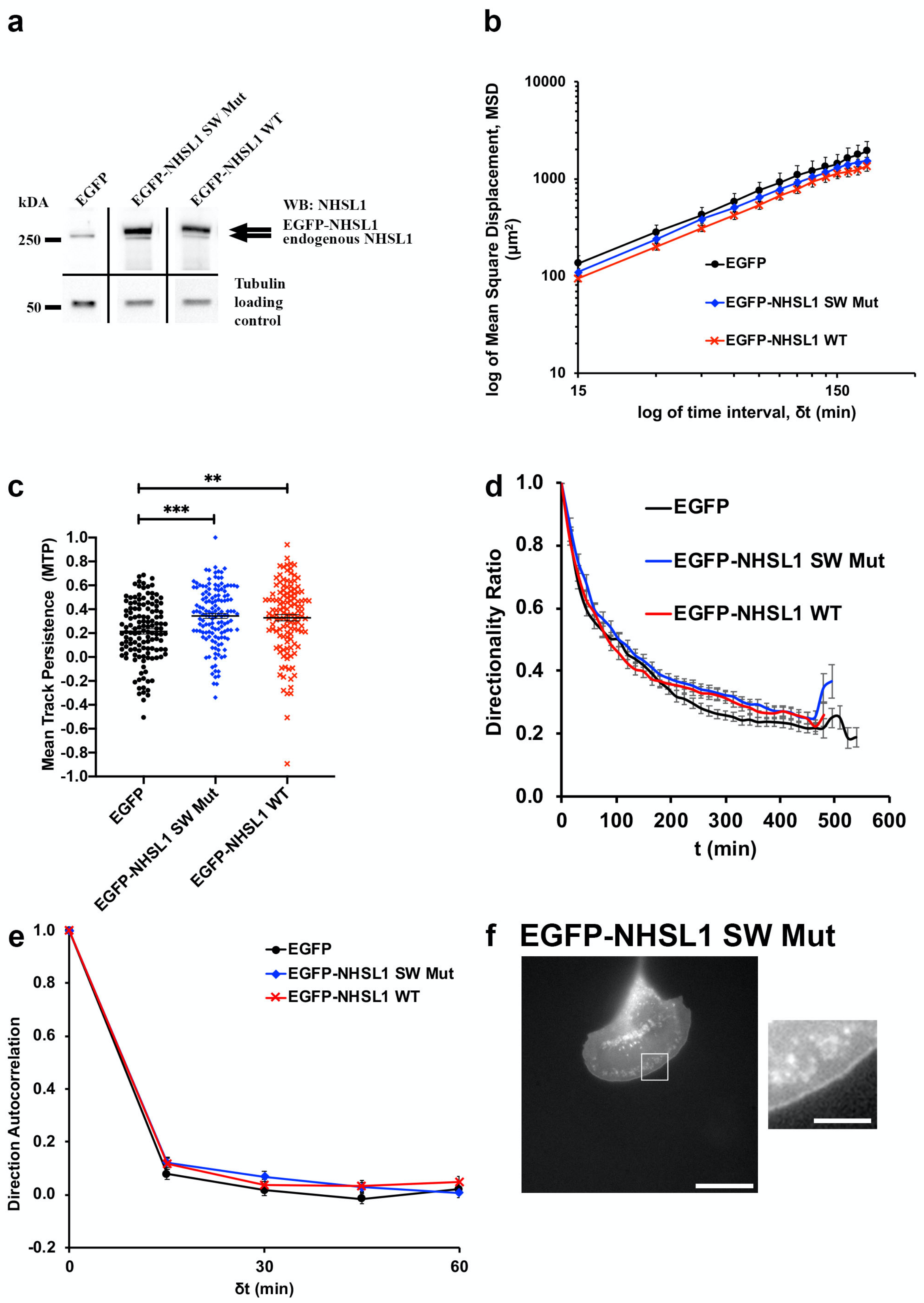

Supplementary Figure 11

Supplementary Figure 11. Effect of NHSL1 overexpression on cell migration persistence.

(a) Western blot showing B16-F1 cells expressing EGFP-NHSL1 wild type (GFP-NHSL1-WT) or the NHSL1 mutant in the Scar/WAVE binding sites (GFP-NHSL1-SW-Mut) or EGFP only as control after selection of the cells with puromycin. The blot was probed with polyclonal antibodies specific for NHSL1 to display the ratio between overexpressed EGFP-NHSL1 (upper band) and endogenous NHSL1 (lower band). Tubulin served as a loading control. Representative blot shown from three independent biological experiments. **(b-e)** Quantification of cell migration persistence of randomly migrating B16-F1 cells expressing either wild type NHSL1 (NHSL1 WT) or the NHSL1 mutant in the Scar/WAVE complex binding sites (NHSL1 SW Mut) or EGFP alone as control plated on fibronectin. **(b)** Mean Square Displacement (MSD) analysis (log-log plot) of data shown in Fig. 6a. **(c)** Mean track persistence (dt = 3, TR = 4; see methods for calculation). One-way ANOVA: $p=0.000087$, $F(2,395)=9.573$; and Dunnett's multiple comparisons test: *** $p=0.0001$; ** $p=0.0011$. **(d)** The directionality ratio is plotted to explore how it changes over the time of the movie. **(e)** Direction autocorrelation is shown, a measure of how the angle of displacement vectors correlate with themselves which is independent of speed. **(b-e)** Results are mean values +/- SEM (error bars); n=106 (control), 104 (NHSL1 WT), 108 (NHSL1 SW mut) cells from four independent biological experiments. **(f)** Still image from live cell imaging showing EGFP-NHSL1 Scar/WAVE binding mutant (EGFP-NHSL1 SW Mut) in B16-F1 cells plated on laminin localises to the leading edge. Representative image from three independent biological repeats. Scale bar: 20 μm. Inset represents a magnified view of the white box. Scale bar in inset: 5 μm. See also related Suppl. Movie 7. Source data are provided as a Source Data file.

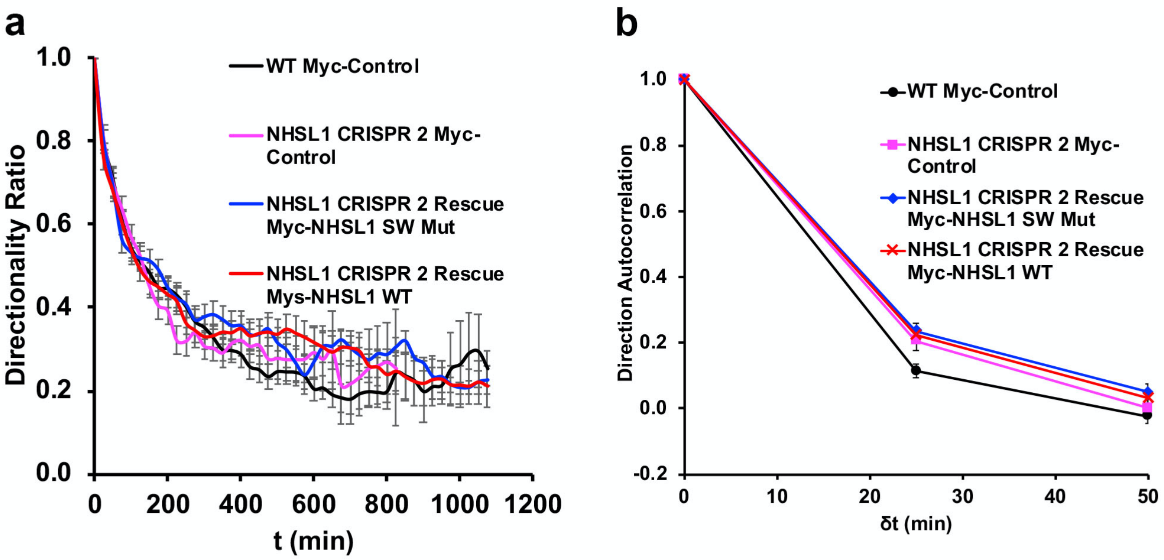

Supplementary Figure 12

Supplementary Figure 12. NHSL1 negatively regulates cell migration.
(a,b) Quantification of persistence of randomly migrating wild type B16-F1 cells expressing Myc alone as control (black circles) or CRISPR 2 cells expressing either the NHSL1 mutant in the Scar/WAVE complex binding sites (NHSL1 SW Mut, blue diamonds) or NHSL1 (NHSL1 WT, red crosses) or Myc alone as control (pink squares) plated on laminin after selection using a bicistronic expression plasmid also conferring resistance to blasticidin to ensure that all cells analysed expressed NHSL1. **(a)** The directionality ratio is plotted to explore how it changes over the time of the movie. **(b)** Direction autocorrelation is shown, a measure of how the angle of displacement vectors correlate with themselves which is independent of speed. **(a-b)** Results are mean values +/- SEM (error bars). n= 102 (wild-type cells Myc only), 55 (CRISPR 2 cells Myc only), 77 (CRISPR 2 Rescue Myc-NHSL1 SW Mut), 72 (CRISPR 2 Rescue Myc-NHSL1 WT) from five independent biological experiments.

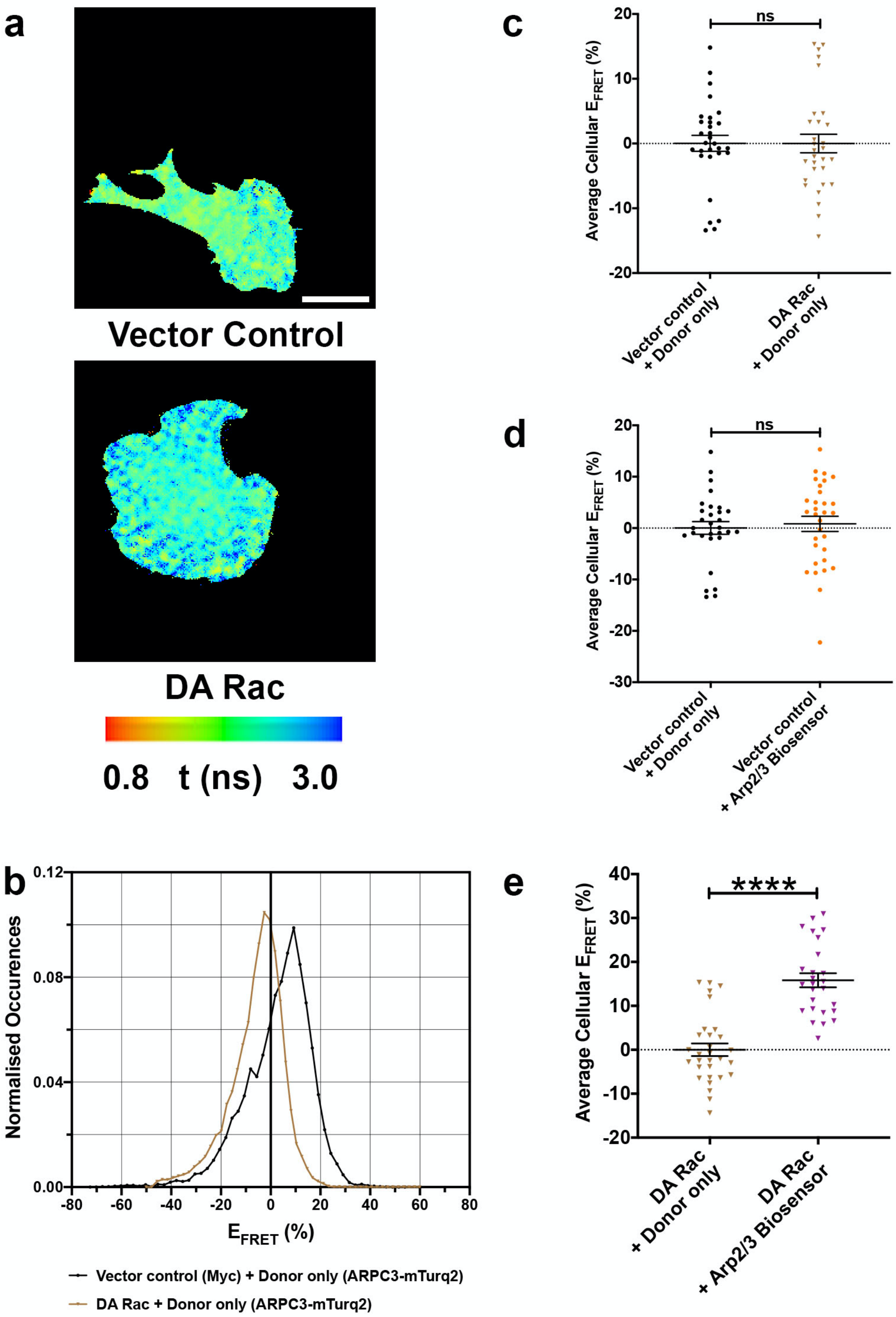

Supplementary Figure 13

Supplementary Figure 13. Active Rac increases Arp2/3 activity - FRET-FLIM controls.
(a) Lifetime images of B16-F1 cells expressing the donor ARPC3-mTurq2 and either empty Myc-control plasmid (vector control) or Myc-tagged dominant active Rac (DA Rac). Warm colours indicate short lifetimes of the donor ARPC3-mTurq2 only as control. Representative images from 4 independent biological repeats are shown. Scale bar: 20 μm. **(b)** FRET efficiency histograms from the same representative B16-F1 cells expressing control Myc plasmid (black circles) and Myc-tagged dominant active Rac (DA Rac) (brown upside-down triangles) cells expressing the donor ARPC3-mTurq2. **(c-e)** Quantification of FRET efficiency controls used to verify that any change in efficiency was a result of real changes in Arp2/3 activity and not skewed by, or an artefact of, the environment, vectors, or the FRET pair itself. The weighted mean average for each cell was calculated from the FRET efficiency histograms and were used instead of the normal mean in order to better represent the true FRET efficiency. Data is shown as weighted average mean ± SEM (error bars). Quantification from 4 independent biological repeats for B16-F1 cells expressing (c) control Myc plasmid and the donor ARPC3-mTurq2 (black circles; n=30 cells) or Myc DA Rac and the donor ARPC3-mTurq2 (brown upside-down triangles; n=30 cells); ns=0.9886; t=0.01436, df=58, Unpaired, two-tailed t-test. (d) control Myc plasmid and the donor ARPC3-mTurq2 (black circles; n=30 cells) or control Myc plasmid and the Arp2/3 biosensor (orange circles; n=31 cells), ns=0.6790, t=0.4158, df=59, Unpaired, two-tailed t-test and (e) Myc DA Rac and the donor ARPC3-mTurq2 (brown upside-down triangles; n=30 cells) or Myc DA Rac and the Arp2/3 biosensor (lilac upside-down triangles; n=26 cells), ****, p=0.000000001, t=7.379, df=54, Unpaired, two-tailed t-test. Source data are provided as a Source Data file.

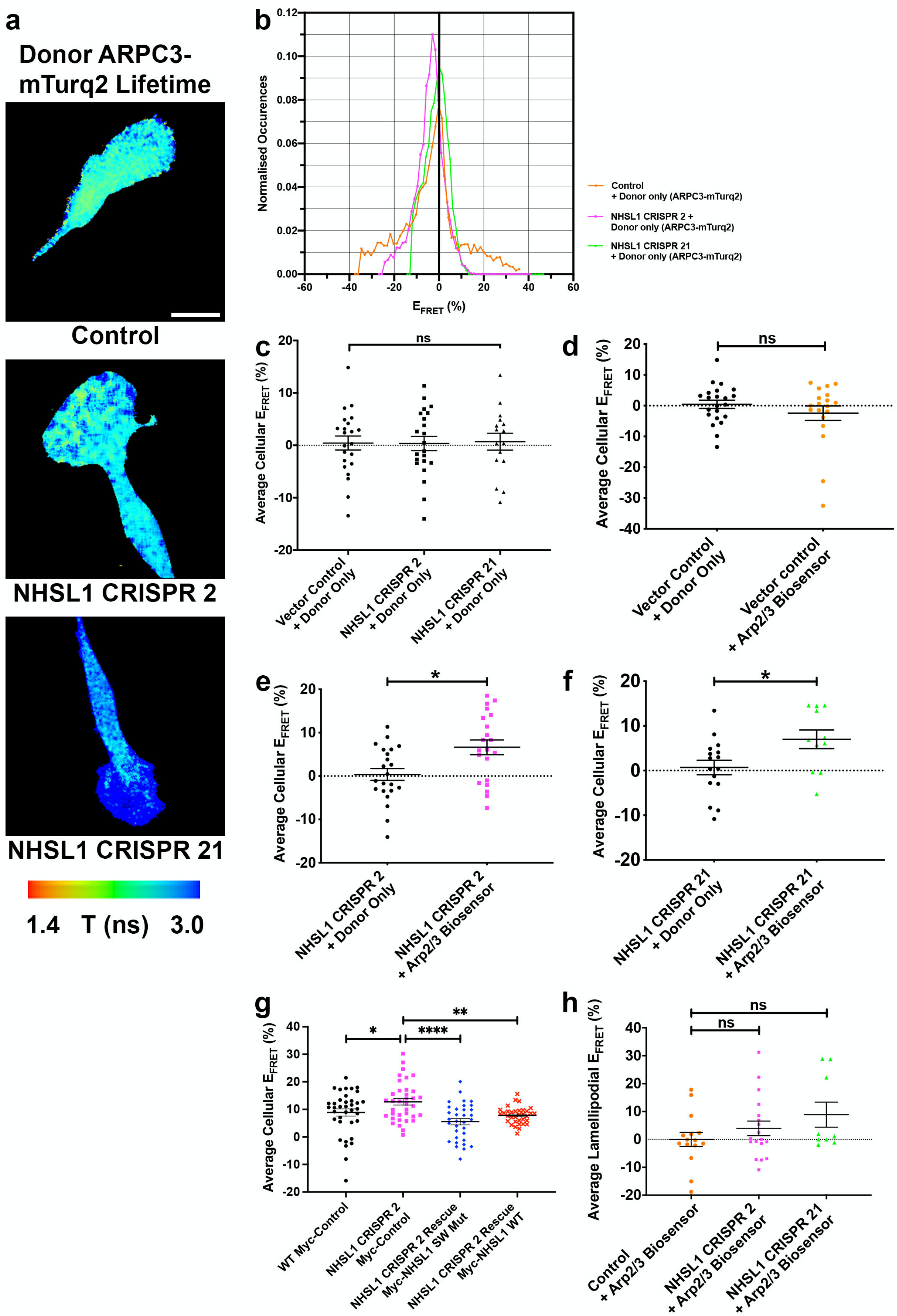

Supplementary Figure 14

Supplementary Figure 14. NHSL1 negatively regulates Arp2/3 activity - FRET-FLIM controls.

(a) Lifetime images of wild type B16-F1, NHSL1 CRISPR clone 2 and NHSL1 CRISPR clone 21 cells expressing the donor ARPC3-mTurq2. Warm colours indicate short lifetimes of the donor mTurq2 only as control. Scale bar: 20 μm. **(b)** FRET efficiency histograms from the same representative wild type B16-F1 (orange circles), NHSL1 CRISPR clone 2 (magenta squares) and NHSL1 CRISPR clone 21 (green triangles) cells expressing the donor ARPC3-mTurq2. **(c-f)** Quantification of FRET efficiency controls used to verify that any change in efficiency was a result of real changes in Arp2/3 activity and not skewed by, or an artefact of, the environment, vectors, or the FRET pair itself for B16-F1 cells expressing (c) donor fluorophore only controls for each biological condition and control Myc plasmid (black circles; n=22 cells), NHSL1 CRISPR2 (black squares; n=22 cells), and NHSL1 CRISPR21 (black triangles; n=16 cells), One-way ANOVA; Tukey's test: p=0.9859, $F(2,57)=0.01420$, control vs. CRISPR2: ns p=0.9991; control vs. CRISPR21: ns p=0.9913; CRISPR2vs. CRISPR21: ns p=0.9853. (d) control Myc plasmid with donor-only fluorophore (black circles; n=22 cells) and donor-acceptor fluorophore (Arp2/3 biosensor; orange circles; n=19 cells), Mann-Whitney test, two tailed: ns: p=0.5957. (e) NHSL1 CRISPR2 with donor-only fluorophore (black circles; n=22 cells) and donor-acceptor fluorophore (Arp2/3 biosensor; magenta squares; n=21 cells), (f) NHSL1 CRISPR21 with donor-only fluorophore (black circles; n=16 cells) and donor-acceptor fluorophore (Arp2/3 biosensor; green triangles; n=11 cells), Mann-Whitney test, two tailed: * p=0.0126. The weighted mean for each cell was calculated from the FRET efficiency histograms and were used instead of the normal mean in order to better represent the true FRET efficiency. Results are an average of the weighted mean ± SEM (error bars). **(g)** Quantification of average cellular FRET efficiency which represents Arp2/3 activity from wild type B16-F1 cells expressing Myc alone as control (black circles; 37 cells) or CRISPR 2 cells expressing either the NHSL1 mutant in the Scar/WAVE complex binding sites (NHSL1 SW Mut, blue diamonds; 33 cells) or NHSL1 (NHSL1 WT, red crosses; 34 cells) or Myc alone as control (pink squares; 36 cells) plated on laminin after selection using a bicistronic blasticidin expression plasmid to ensure that all cells analysed expressed NHSL1. Data points are the weighted average means for each cell ± SEM (error bars), calculated from the FRET efficiency histograms. One-way ANOVA: p=0.0001; $F(3,136)=7.421$; and Dunnett's multiple comparisons test: CRISPR2 vs. WT control: *, p=0.0324; NHSL1 CRISPR2 vs. Rescue Myc-NHSL1 SW Mut: ****, p<0.0001; NHSL1 CRISPR2 vs. Rescue Myc-NHSL1 WT: **, p=0.0052. **(h)** Quantification of FRET efficiency which represents Arp2/3 activity in the approximate area of the lamellipodium from control: (orange circles; n=15 cells); NHSL1 CRISPR 2: (magenta squares; n=18 cells); NHSL1 CRISPR 21: (green triangles; n=9 cells). Data points are the weighted mean for each cell calculated from the FRET efficiency histograms. Results: average weighted mean ± SEM (error bars), Kruskal-Wallis: p=0.2966, Dunn's test: Control vs. CRISPR 2: ns, p=0.6914 and Control vs. CRISPR 21: ns, p=0.2502. (a-h) Representative images or quantification from four (a-f,h) or five (g) independent biological repeats. Source data are provided as a Source Data file.

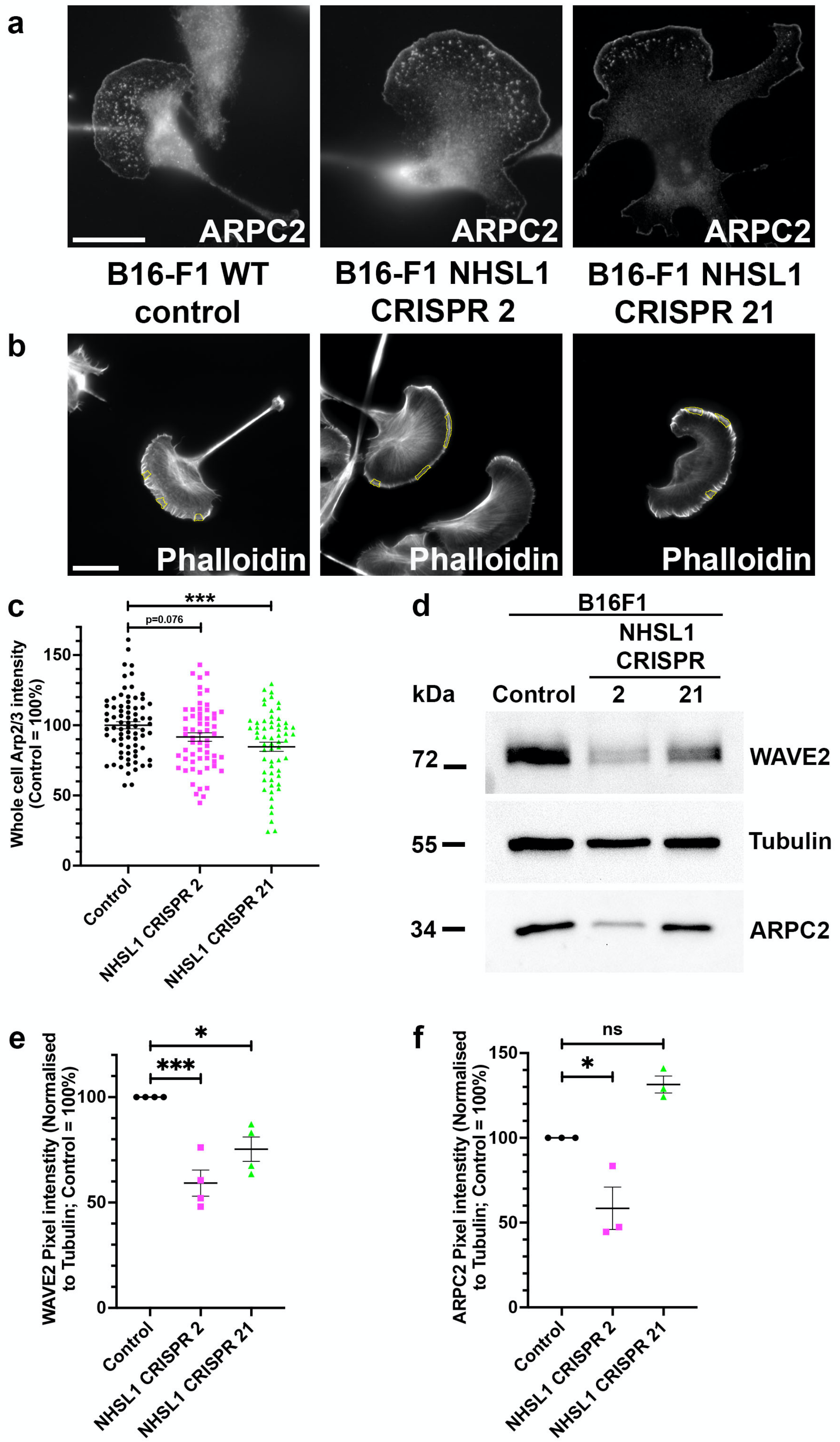

Supplementary Figure 15

Supplementary Figure 15. NHSL1 knockout reduces whole cell Scar/WAVE2, ArpC2, and F-actin levels.
(a,b) Example images for immunofluorescence analysis of (a) ARPC2 for whole cell Arp2/3 complex intensity quantification in (c) and Fig. 8d and (b) F-actin (Alexa488-Phalloidin) in Fig. 8e. Representative images shown from (a) three or (b) five independent biological experiments. (a,b) Scale bar: 20 μm **(c)** Quantification of whole cell Arp2/3 intensity after background subtraction. One-way ANOVA: p=0.0010; F(2,192)=7.202, Dunnett's multiple comparisons test: *** p=0.0004; ns p=0.0758; in wild-type (n=75 cells, control), NHSL1 CRISPR 2 (n=60 cells) and NHSL1 CRISPR 21 (n=60 cells) B16-F1 cells plated on laminin and stained with anti-ARPC2 (subunit of Arp2//3 complex) antibodies; Results are mean ± SEM (error bars), three independent biological repeats. **(d)** Western blot comparing Scar/WAVE2 and ARPC2 expression levels in wild type control B16-F1 cell lysates with NHSL1 CRISPR 2 or 21 cell lysates. Beta-Tubulin serves as the loading control. Representative blot shown from three independent biological experiments. **(e,f)** Quantification of the western blot in (d) for Scar/WAVE2 in (e) and for ARPC2 in (f) normalised to the Tubulin loading control; One-way ANOVA: (e) p=0.0008; F(2,9)=17.63; (f) p=0.0017; F(2,6)=22.19; Dunnett's multiple comparisons test: control vs. CRISPR 2: (e) *** p=0.0004; (f) * p=0.0163; control vs. CRISPR 21(e) * p=0.0110; (f) ns p=0.0502. **(e,f)** Results are mean values +/- SEM (error bars) from four (e) or three (f) independent biological experiments.

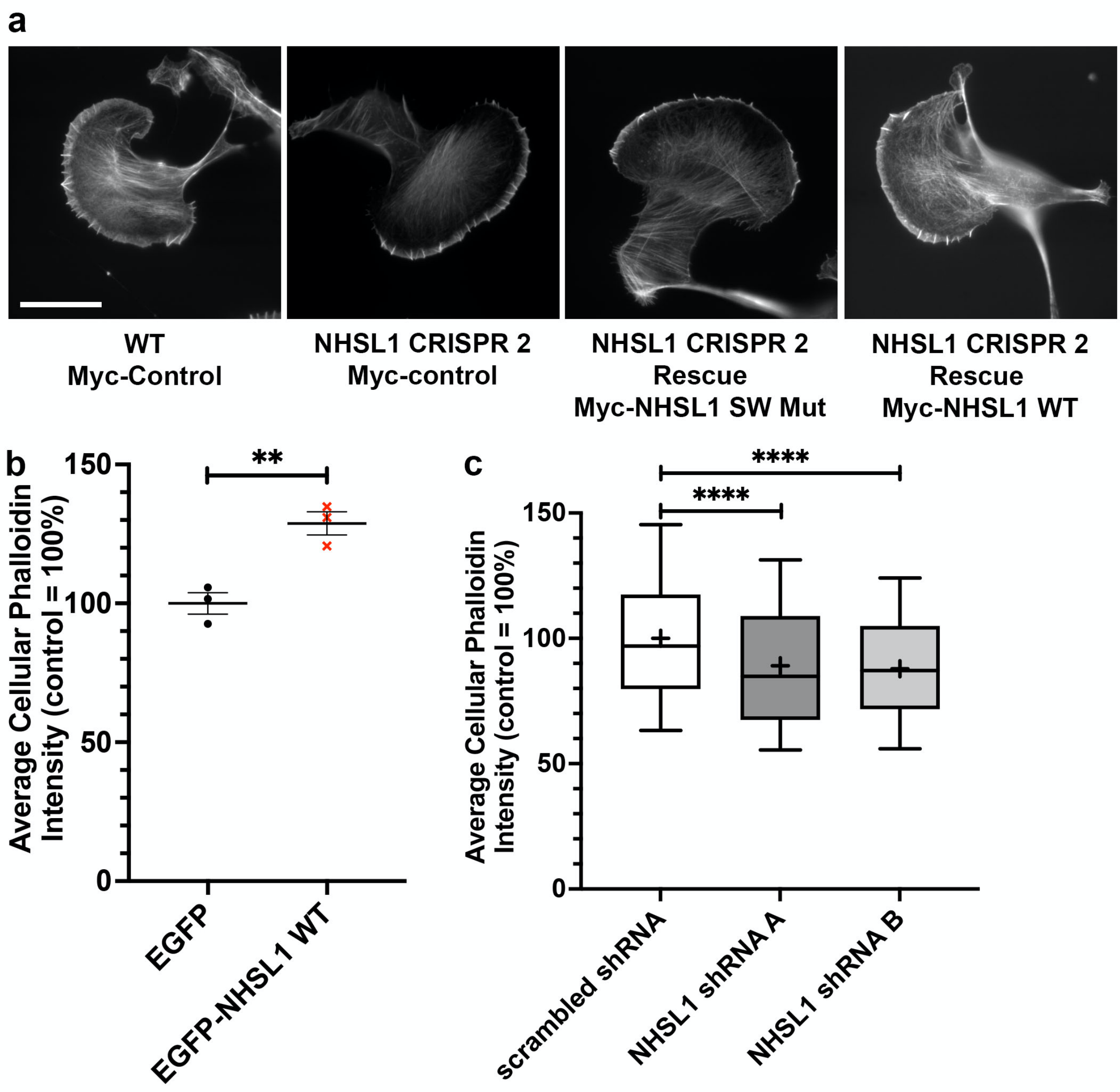

Supplementary Figure 16

Supplementary Figure 16. NHSL1 knockout reduces whole cell F-actin levels.
(a) Example images for whole cell F-actin (Alexa488-Phalloidin) intensity quantification in Fig. 8c. **(b and c)** Quantification of F-Actin content in HEK293 cells that were transfected with (b) EGFP or EGFP-NHSL1 and (c) control shRNA or two independent NHSL1-specific shRNA's (see Fig. S5c), which were puromycin selected and replated for 1 hour on fibronectin coated coverslips. Three independent biological experiments were used for this quantification. (b) EGFP-NHSL1 overexpression increased F-Actin content by 28.8±5.7% SEM, EGFP (black circles): 134 cells total in all 3 experiments; EGFP-NHSL1 (red crosses): 128 cells total in all 3 experiments; Each dot or cross represents the mean of one independent biological repeats; t=5.054, df=4, **, p=0.0072, unpaired, two-tailed t-test. (c) Box and whiskers plot: Box 25 and 75 percentile; whiskers: 10 and 90 percentile; line: median; cross: mean; NHSL1 knockdown decreased F-Actin content by 10.9±0.7% SEM (shRNA A) or 12.2±0.7% SEM (shRNA B), *** = $p \leq 0.001$, Kruskal-Wallis test: Kruskal Wallis statistics: 164.3; $p=2.1 \times 10^{-36}$ and Dunn's multiple comparisons test: scrambled shRNA vs. shRNA A: ****, $p=2.3 \times 10^{-30}$ and scrambled shRNA vs. shRNA B: ****, $p=7.6 \times 10^{-28}$; scrambled shRNA: 1811 cells; shRNA A: 2196 cells; shRNA B: 2148 cells from three independent biological repeats.

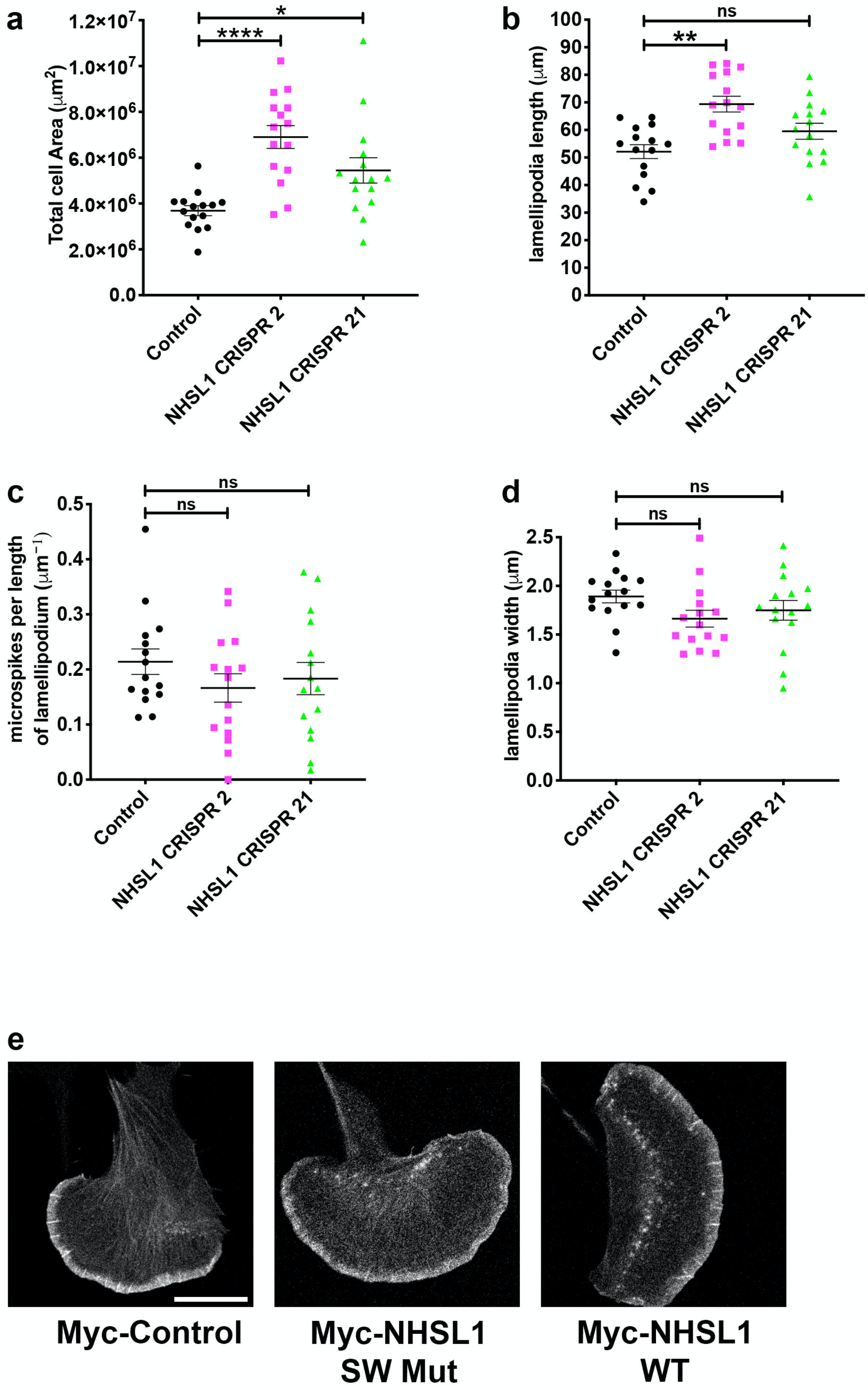

Supplementary Figure 17

Supplementary Figure 17. NHSL1 negatively regulates cell area and lamellipodial length.

(a,b) Quantification of total cell area and lamellipodia length from first frame of a movie of LifeAct-EGFP expressing wild type B16-F1 or NHSL1 CRISPR 2 or 21 cells plated on laminin. (a) Quantification of total cell area, ****, p<0.0001; *, p=0.0160; F(2,42)=12.86. Scale bar: 20 μm. (b) Quantification of lamellipodia length: The length was quantified using the Fiji plugin "measure_ROI" (http://www.optinav.info/Measure-Roi.htm: Measure_Roi_Curve.java) which measures the length of curved objects (see methods for details). See Fig. 8a for an example B16-F1 cell shown indicating the definition of length. ****, p<0.0001; ns, p=0.1193; F(2,42)=9.739. Results are mean ± SEM (error bars), four independent biological repeats, n=15 cells. One-way ANOVA, Dunnett's multiple comparisons test. **(c)** Quantification of number of microspikes per length of lamellipodium; One-way ANOVA: F(2,42)=0.8509; Dunnett's multiple comparisons test: ns (CRISPR2), p=0.3400; ns(CRISPR21), p=0.6206; in wild type (control), NHSL1 CRISPR 2, and NHSL1 CRISPR 21 B16-F1 cells plated on laminin expressing LifeAct-EGFP; Results are mean ± SEM (error bars), four independent biological repeats, n = 15 cells. **(d)** Quantification of lamellipodia width, ns (CRISPR2), p=0.1173; ns(CRISPR21), p=0.4013; F(2,42)=1.89, One-way ANOVA, Dunnett's multiple comparisons test. Four independent biological repeats, n = 15 cells. Source data are provided as a Source Data file. **(e)** Example images for lamellipodia LifeAct-EGFP intensity quantification in Fig. 8f. Scale bar: 20 μm.

a

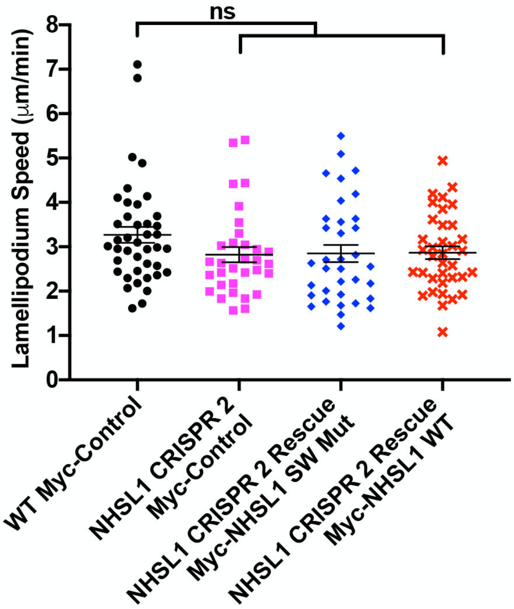

b

% AA identity	hsNHS-Uniprot-Q6T4R5	hsNHSL1-Uniprot-Q5SYE7	hsNHSL2-Uniprot-Q5HYW2
hsNHS-Uniprot-Q6T4R5	100.0	30.3	31.7
hsNHSL1-Uniprot-Q5SYE7	30.3	100.0	25.1
hsNHSL2-Uniprot-Q5HYW2	31.7	25.1	100.0

c **NHSL1 Abi binding site 2**

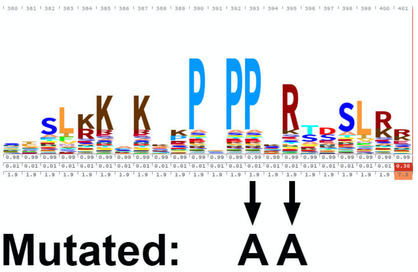

d **NHSL1 Abi binding site 3**

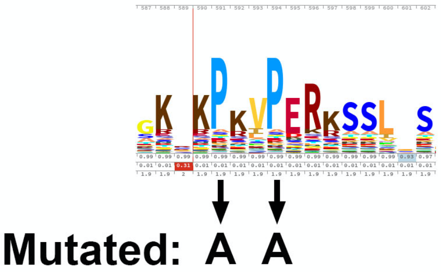

Supplementary Figure 18

Supplementary Figure 18. Analysis of segmented lamellipodium speed and two Abi binding sites are conserved in the NHS protein family.

(**a**) Quantification of the average speed of the centroid of the segmented lamellipodium from a wild type cell (WT), NHSL1 knockout cell (NHSL1 CRISPR2), and NHSL1 knockout cells rescued with a wild type NHSL1 construct (NHSL1 CRISPR 2 Rescue Myc-NHSL1 WT) and with a mutant NHSL1 construct (NHSL1 CRISPR 2 Rescue Myc-NHSL1 SW Mut). Results are mean ± SEM; n = 41, 32, 35, and 36 for the respective conditions (left to right on x-axis), from six independent biological repeats. One-way ANOVA: p=0.1948; F(3,140)=1.589; and Tukey's multiple comparisons test: Control vs. CRISPR 2: p=0.2755; Control vs. NHSL1 SW Mut Rescue: p=0.3091; Control vs. NHSL1 WT Rescue: p=0.3399; CRISPR 2 vs. NHSL1 SW Mut Rescue: p=0.9996; CRISPR 2 vs. NHSL1 WT Rescue: p=0.9981; NHSL1 SW Mut Rescue vs. NHSL1 WT Rescue: p=0.9999. See Suppl. Movie 8. **(b)** The conservation on the amino acid level in the NHS protein family is shown as percent amino acid identity as calculated from a Clustal Omega alignment (DNASTAR MegAlignPro) from the UniProt canonical amino acid sequences of each NHS family member. The UniProt identifier of each the sequence of each NHS family member used is displayed in the table. **(c,d)** HHM logo of conservation of Abi SH3 binding sites in the NHS protein family (from PFAM PF15273). Source data are provided as a Source Data file.
