# Supplementary material for: Nance-Horan Syndrome-like 1 protein negatively regulates Scar/WAVE-Arp2/3 activity and inhibits lamellipodia stability and cell migration
Source: Nat Commun. 2021 Sep 28;12:5687. doi: 10.1038/s41467-021-25916-6 (PMC8478917; doi:10.1038/s41467-021-25916-6)
Supplement: Supplementary file 3 — Description of Additional Supplementary Files [file 41467_2021_25916_MOESM3_ESM.pdf]

Description of Additional Supplementary Files

File Name: Supplementary Data 1
Description: The sequences of all primers used in this study are supplied here in this Supplementary Data 1 file.

File Name: Supplementary Movie 1
Description: **NHSL1 localises to the very edge of lamellipodia.**

This movie shows that either N- or C- terminally EGFP-tagged NHSL1 expressed in B16-F1 cells plated on laminin localises to the very edge of protruding lamellipodia. Representative movie shown from three independent biological repeats. Cells were imaged every 10 seconds for the indicated times in minutes and seconds by wide-field time-lapse video microscopy using an IX 81 microscope (Olympus). Scale bar: 20 μm.

File Name: Supplementary Movie 2
Description: **EGFP-NHSL1 localisation to the leading edge is not due to space filling of the fluorescent protein.**

This movie shows EGFP-NHSL1 co-expressed with mScarlet-I in B16-F1 cells to control for detection at the leading edge due to space filling of the fluorescent protein. The subtracted movie is generated from the subtraction of the signal of the mScarlet-I from the NHSL1-EGFP signal for each frame. Representative movie shown from three independent biological repeats. Cells were imaged every 10 seconds for the indicated times in minutes and seconds by wide-field time-lapse video microscopy using an IX 81 microscope (Olympus). Scale bar: 20 μm.

File Name: Supplementary Movie 3
Description: **NHSL1 co-localise with Lpd at the edge of lamellipodia.**

This movie shows that NHSL1-EGFP co-expressed with mScarlet-I-tagged Lpd co-localise together at the edge of lamellipodia in B16-F1 cells plated on laminin. Representative movie shown from three independent experiments. Cells were imaged every 10 seconds for the

indicated times in minutes and seconds by wide-field time-lapse video microscopy using an IX 81 microscope (Olympus). Scale bar: 20 μm.

File Name: Supplementary Movie 4
Description: **NHSL1 knockdown increases cell migration.**

This movie shows that directional migration into a scratch wound was significantly increased in MCF10A normal breast epithelial cells in which NHSL1 expression had been knocked-down for both shRNAs tested. Confluent monolayers were scratch-wounded and imaged every 5 min for 12 hr by time-lapse phase contrast wide-field microscopy using an IX 81 microscope (Olympus). Scale bar: 300 μm.

File Name: Supplementary Movie 5
Description: **Specific NHSL1 fragments localise to the leading edge and to vesicular structures.**

These movies show the localisation of four EGFP-tagged fragments covering the entire length of NHSL1 expressed in B16-F1 cells. Fragment 2 and 3 localise to the very edge of lamellipodia similar to full length NHSL1 (see video S1). EGFP alone served as the negative control. In addition, fragment 1 and 3 were detected at vesicular structures.
Representative movies shown from three independent experiments. Cells were imaged every 10 seconds for the indicated times in minutes and seconds by wide-field time-lapse video microscopy using an IX 81 microscope (Olympus). Scale bar: 20 μm.

File Name: Supplementary Movie 6
Description: **NHSL1 co-localise with components of the Scar/WAVE complex.**

These movies show NHSL1-EGFP co-expressed with mScarlet-I-tagged Abi1 or Nap1 co-localise at the leading edge of cells in B16-F1 cells plated on laminin. Representative movies shown from three independent experiments. Cells were imaged every 10 seconds for the

indicated times in minutes and seconds by wide-field time-lapse video microscopy using an IX 81 microscope (Olympus). Scale bar: 20 μm.

File Name: Supplementary Movie 7
Description: **The NHSL1 Scar/WAVE binding mutant localises to the very edge of lamellipodia.**

This movie shows that the NHSL1 Scar/WAVE binding mutant localises to the very edge of lamellipodia like wild type EGFP-NHSL1. Representative movie shown from three independent experiments. Cells were imaged every 10 seconds for the indicated times in minutes and seconds by wide-field time-lapse video microscopy using an IX 81 microscope (Olympus). Scale bar: 20 μm.

File Name: Supplementary Movie 8
Description: **NHSL1 reduces actin retrograde flow speed and F-actin assembly rate.**

This movie shows that NHSL1 reduces actin retrograde flow speed and F-actin assembly rate. Shown is a segmented lamellipodium from a wild type cell (WT), NHSL1 knockout cell (NHSL1 CRISPR2), and NHSL1 knockout cells rescued with a wildtype NHSL1 construct (NHSL1 CRISPR 2 Rescue Myc-NHSL1 WT) and with a mutant NHSL1 construct (NHSL1 CRISPR 2 Rescue Myc-NHSL1 SW Mut). Left panels show segmented Airyscan movies; right panels show the corresponding movies from the PIV interpolation. Each colourmap has the same range (0-3 μm/min) and distance between vector arrows (1 μm); warmer colours represent faster flow, vector arrows are all of unit length. Representative movie shown from six independent biological repeats. Cells were imaged every 3.22 seconds for the indicated times in minutes and seconds by confocal time-lapse video microscopy using a Zeiss Airyscan microscope (Zeiss). Scale bar: 10 μm.

File Name: Supplementary Movie 9

Description: **NHSL1 CRISPR cells display a lamellipodium which is less interrupted by retractions.**

This movie shows that NHSL1 CRISPR cells displayed a lamellipodium which is less interrupted by retractions. B16-F1 cells were transfected with LifeAct-EGFP and plated onto laminin. Representative movie shown from four independent experiments. Cells were imaged every 5 seconds for the indicated times in minutes and seconds by confocal time-lapse video microscopy using a Zeiss Airyscan microscope (Zeiss). The cell outline was automatically segmented in each frame of the movies, protrusion vectors at each pixel along the cell edge calculated (red arrows shown in lower movie panels), the cell edge binarised and areas of protrusions along the cell edge indicated by a blue line and areas of retraction by a red line (shown in upper movie panels). Scale bar: 20 μm.

File Name: Supplementary Movie 10
Description: **Cells overexpressing Myc-NHSL1 display a lamellipodium which is more interrupted by retractions.**

This movie shows that the protruding lamellipodium is more interrupted by retractions in cells overexpressing Myc-NHSL1. B16-F1 cells were transfected with Myc-NHSL1-IRES-Puro-T2A-LifeAct-EGFP or empty Myc-IRES-Puro-T2A-LifeAct-EGFP, selected with puromycin and plated onto laminin. Representative movie shown from four independent experiments. Cells were imaged every 5 seconds for the indicated times in minutes and seconds by confocal time-lapse video microscopy using a Zeiss Airyscan microscope (Zeiss). The cell outline was automatically segmented in each frame of the movies, protrusion vectors at each pixel along the cell edge calculated (red arrows shown in lower movie panels), the cell edge binarised and areas of protrusions along the cell edge indicated by a blue line and areas of retraction by a red line (shown in upper movie panels). Scale bar: 20 μm.

File Name: Supplementary Software 1

Description: This zip file contains the additional Matlab codes and instructions for "**Quantification of lamellipodia protrusion speed"** and for **"Analysis of length distribution of lamellipodia"** to be used in conjunction with the Windowing-protrusion analysis package from the Danuser lab which can be downloaded at: https://github.com/DanuserLab/Windowing-Protrusion
