# Supplementary material for: Nance-Horan Syndrome-like 1 protein negatively regulates Scar/WAVE-Arp2/3 activity and inhibits lamellipodia stability and cell migration
Source: Nat Commun. 2021 Sep 28;12:5687. doi: 10.1038/s41467-021-25916-6 (PMC8478917; doi:10.1038/s41467-021-25916-6)
Supplement: Supplementary file 4 — Supplementary Data 1 [file 41467_2021_25916_MOESM4_ESM.docx]

| Supplementary Data 1: Complete list of all primers | | |
| --- | --- | --- |
| Used to generate | Sequence 5’-3’ | Additional information |
| human NHSL1d full length cDNA | GGG GAC AAG TTT GTA CAA AAA AGC AGG CTT CGC CAC CAT GGT GGT CTT CAT TAA TGC | AttB1  Into pDONR207 |
| human NHSL1d full length cDNA | GGG GAC CAC TTT GTA CAA GAA AGC TGG GTG CTA ACT CTC CTC GCT CAG | AttB2  Into pDONR207 |
| 5' in situ for mouse NHSL1 into pBS | cgggatcctctagccagtccgaatcc | BamH I |
| 3' new in situ for mouse NHSL1 into pBS | ggaattcctgactcttcctgtgtgg | EcoRI |
| shRNA-A for human NHSL1 into pLL3.7Puro | TGCCCATTCTGTGATGATTATTCAAGAGATAATCATCACAGAATGGGCTTTTTggaaagaattcg |  |
| shRNA-A for human NHSL1 into pLL3.7Puro | TCGAcgaattctttccAAAAAGCCCATTCTGTGATGATTATCTCTTGAATAATCATCACAGAATGGGCA |  |
| shRNA-B for human NHSL1 into pLL3.7Puro | TGGATAAATCCCTATCAAGATTCAAGAGATCTTGATAGGGATTTATCCTTTTTggaaagaattcg |  |
| shRNA-B for human NHSL1 into pLL3.7Puro | TCGAcgaattctttccAAAAAGGATAAATCCCTATCAAGATCTCTTGAATCTTGATAGGGATTTATCCA |  |
| NHSL1 GST-1 | cgggATccgcAggcTTcgccAccATgg | pGEX-6P1 |
| NHSL1 GST-1 | cggAATTcTcAcATggAAggTggTAcgAcc | pGEX-6P1 |
| NHSL1 GST-2 | cgggATccAggAgAATcAgggcAcAgAAggg | pGEX-6P1 |
| NHSL1 GST-2 | cggAATTcTcAATAcggcATAgcTTgggAcc | pGEX-6P1 |
| NHSL1 GST-3 | cg gAA TTc TcA Agg AgA AAc cAc AcA cgc Tgg | pGEX-6P1 |
| NHSL1 GST-3 | cg ggA Tcc cAT gcA ATc TAc Tcc Acc Agc | pGEX-6P1 |
| NHSL1 GST-4 | cgggATccAATTgTAgAAAcAAccTggcc | pGEX-6P1 |
| NHSL1 GST-4 | cggAATTcTcATgTgTcAcTcTgcgATggcg | pGEX-6P1 |
| NHSL1 GST-5 | ggagaattccatcaatcaggtgg | pGEX-6P1 |
| NHSL1 GST-6 | gattggtgcagatctcctccc | pGEX-6P1 |
| NHSL1 GST-6 | gagaattctaatctttctgcggaggtgg | pGEX-6P1 |
| NHSL1 GST-7 | gaagatctgattttgcagtggagccc | pGEX-6P1 |
| NHSL1 GST-7 | ctttcgaattcatctctgaatcg | pGEX-6P1 |
| NHSL1 GST-8 | gaagatctagcatccgaaagagcagc | pGEX-6P1 |
| NHSL1 GST-8 | gagaattcttggccctaactctcc | pGEX-6P1 |
|  |  |  |
| Mutation of Abi1 SH3-1 binding site in NHSL1 | P-GGAGTTGCTAAATGCGcCTTgAGGGcGGATGCCgcTCCATTACCTG |  |
| Mutation of Abi1 SH3-2 binding site in NHSL1 | P-GGAGTCTGTCCGcGAGGcTGGCAGGGcAGcCTTCgcTGCTTTCTTC |  |
| Mutation of Abi1 SH3-3 binding site in NHSL1 | c AAg gcc AAg gTA gcA gAA Agg Aag Tcc TcT cTg ATA TcT TcA g |  |
| Mutation of Abi1 SH3-3 binding site in NHSL1 | cT TTc Tgc Tac cTT ggc cTT ggg cTT ccc cTT ccc Gtt Tgc Tgg |  |
| mouse NHSL1 CRISPR KO exon 2 sgRNA1 fwd | caccgTCGACTCTCCTCGTCCAAGT | BbsI |
| mouse NHSL1 CRISPR KO exon 2 sgRNA1 rev | aaacACTTGGACGAGGAGAGTCGAc | BbsI |
| mouse NHSL1 CRISPR KO exon 2 sgRNA2 fwd | caccgCTGTCCACTACACGGCACCA | BbsI |
| mouse NHSL1 CRISPR KO exon 2 sgRNA2 rev | aaacTGGTGCCGTGTAGTGGACAGc | BbsI |
| 5' homology arm of mmNHSL1-KO into pCRISPR-donor | gccagtGAATTCttgaaatgctgctgatgaatgc |  |
| 5' homology arm of mmNHSL1-KO into pCRISPR-donor | gtggaaAGATCTaaTGGAAACCGctacagagaaaaaga | BglII |
| 3' homology arm of mmNHSL1-KO into pCRISPR-donor | cgcGGATCCGCACCATGGCATCAGCAAGAGA |  |
| 3' homology arm of mmNHSL1-KO into pCRISPR-donor | aagcttgcatgccgaacaaatcacttagcaaatggc |  |
| for genotyping of WT allele NHSL1 CRISPR KO | ctctcagtgtattccaggct |  |
| for genotyping of WT allele NHSL1 CRISPR KO | gggttctttgaaggcggacg |  |
| for genotyping of left arm integration of KO allele NHSL1 CRISPR | aggtctacactctgccgttctggg |  |
| for genotyping of left arm integration of KO allele NHSL1 CRISPR | CGGCCGCtgTCTAGATTTTTGAa |  |
| for genotyping of right arm integration of KO allele NHSL1 CRISPR | CTAGCGGGGGAGGGACG |  |
| for genotyping of right arm integration of KO allele NHSL1 CRISPR | accgttatgaagtagcaatgaaaa |  |
| amplify hsNHSL1 full length w/o stop codon into pENTR3C | ggaAGATCTgccaccATGGTGGTCTTCATTAATGC | Bgl II |
| amplify hsNHSL1 full length w/o stop codon into pENTR3C | ccgGAATTCgcACTCTCCTCGCTCAGAGAACCGCC | EcoRI |
| hsNHSL1 fragment 1 into pENTR3C | gccGAATTCaccATGGTGGTCTTCATTAATGC | EcoRI |
| hsNHSL1 fragment 1 into pENTR3C | TGGGTCTAGAttaGTGGTCTCTGGATTTTATCCTTG | XbaI |
| hsNHSL1 fragment 2 into pENTR3C | gccGAATTCCTCATCTCCAGGCATGCTGTG | EcoRI |
| hsNHSL1 fragment 2 into pENTR3C | TGGGTCTAGAttaAGTGCCACTTCCTTCAGTAGAAG | XbaI |
| hsNHSL1 fragment 3 into pENTR3C | gccGAATTCATGAAGAAGCTGGATCCAGCC | EcoRI |
| hsNHSL1 fragment 3 into pENTR3C | ctagTCTAGAttaCTCTGCGGGCTCCACTGC | XbaI |
| hsNHSL1 fragment 4 into pENTR3C | gccGAATTCAACGTGAGCGAAGCCCTCC | EcoRI |
| hsNHSL1 fragment 4 into pENTR3C | tgggTCTAGActaactctcctcgctcagag | XbaI |
|  |  |  |
| NHSL1 fragment 23-1 w/o stop into pENTR-3C | tgggtctagattaGCCATCGTGGTCCTCAGAATAC | XbaI |
| NHSL1 fragment 23-2 w/o stop into pENTR-3C | gccGAATTCTACTGTGCATCTGTGCACACTG | EcoRI |
| NHSL1 fragment 23-2 w/o stop into pENTR-3C | tgggTCTAGAttaCAGGGAGTAGACATTGGGGG | XbaI |
| NHSL1 fragment 23-3 w/o stop into pENTR-3C | gccGAATTCTGCGGGGCCACGCCATCG | EcoRI |
| NHSL1 fragment 23-3 w/o stop into pENTR-3C | tgggTCTAGAttaTCCTTCAGTAGAAGTACTAGAAG | XbaI |
| NHSL1 fragment 23-4 w/o stop into pENTR-3C | gccGAATTCAGTGGCACTATGAAGAAGCTGG | EcoRI |
| NHSL1 fragment 23-4 w/o stop into pENTR-3C | TGGGTCTAGAttaCAATGCTTCCGTGGTTATCAGG | XbaI |
| NHSL1 fragment 23-5 w/o stop into pENTR-3C | gccGAATTCCAGATGGTGCAGTTGAGGCC | EcoRI |
| NHSL1 fragment 23-6 w/o stop into pENTR-3C | gccGAATTCCCCCAAGATGTGGATGGCAAG | EcoRI |
| NHSL1 fragment 23-6 w/o stop into pENTR-3C | TGGGTCTAGAttaGCGGAGGGAGTCTGTCCGG | XbaI |
| NHSL1 fragment 23-7 w/o stop into pENTR-3C | gccGAATTCAGGATTCCCAAGAAGAGCAGC | EcoRI |
| NHSL1 fragment 23-7 w/o stop into pENTR-3C | TGGGTCTAGAttaCTTTGGTTTGACTGAACCACCG | XbaI |
| NHSL1 fragment 23-8 w/o stop into pENTR-3C | gccGAATTCATCATGTCACCAGAGAAGTCAC | EcoRI |
| NHSL1 fragment 23-8 w/o stop into pENTR-3C | TGGGTCTAGAttaGCACCAATCAGGTGGGGAGC | XbaI |
| NHSL1 fragment 23-9 w/o stop into pENTR-3C | gccGAATTCTGCCTTTCTCCTCCCCGCC | EcoRI |
| NHSL1 fragment 23-9 w/o stop into pENTR-3C | TGGGTCTAGAttaAGGCGTCGGAAGCGAGCTTG | XbaI |
| Q-PCR primer for GusB | ACCACACCCAGCCAATAAAG | IDT:  Mm.PT.39a.22214848 |
| Q-PCR primer for GusB | AGCAATGGTACCGGCAG | IDT:  Mm.PT.39a.22214848 |
| Q-PCR primer for NHSL1 | TCTTCATCTTGATAATCGTCGCA | IDT:  Mm.PT.58.41480072 |
| Q-PCR primer for NHSL1 | CAAGCTCAACCTCAAATCAGTG | IDT:  Mm.PT.58.41480072 |
|  |  |  |
|  |  |  |
| Hifi cloning of DEST for pCAG-DEST-mEGFP-2A-Puro | CTGCAGTCGACGGTACcAACAAGTTTGTACAAAAAAGCTG | restores KpnI |
| Hifi cloning of DEST for pCAG-DEST-mEGFP-2A-Puro | tggtggctGCATGCACGCGATCAACC |  |
| Hifi cloning of mEGFP for pCAG-DEST-mEGFP-2A-Puro | GTGCATGCagccaccatggtgagcaagg |  |
| Hifi cloning of mEGFP for pCAG-DEST-mEGFP-2A-Puro | CTCtccgcttcccttgtacagctcgtccatgc |  |
| Hifi cloning of T2A-Puro for pCAG-DEST-mEGFP-2A-Puro | caagggaagcggaGAGGGCAGAGGAAGTCTTC |  |
| Hifi cloning of T2A-Puro for pCAG-DEST-mEGFP-2A-Puro | TGCACCTGAGGAGTGCGGCCGCtcaggcaccgggcttgcg | restores NotI |
| Hifi cloning of DEST for pCAG-mEGFP-DEST-IRES-Puro | cgagctgtacaagGTACCAACAAGTTTGTACAAAAAAGCTG | restores KpnI |
| Hifi cloning of DEST for pCAG-mEGFP-DEST-IRES-Puro | ccgctacGCATGCACGCGATCAACC |  |
| Hifi cloning of IRES-Puro for pCAG-mEGFP-DEST-IRES-Puro | GTGCATGCgtagcggccgcaaattccg |  |
| Hifi cloning of IRES-Puro for pCAG-mEGFP-DEST-IRES-Puro | TGCACCTGAGGAGTGCGGCCGCtcaggcaccgggcttgcg | restores NotI |
| Hifi cloning of pCAG-Myc-DEST-IRES-Puro | GGTTATTGTGCTGTCTCATCATTTTGGCAAAGAATTCgCCACCATGGAGCAGAAGCTG | EcoRI |
| Hifi cloning of pCAG-Myc-DEST-IRES-Puro | ggaatttgcggccgctacACCACTTTGTACAAGAAAGCTG |  |
| Hifi cloning of pCAG-Myc-DEST-IRES-Puro | AGCTTTCTTGTACAAAGTGGTgtagcggccgcaaattccg |  |
| Hifi cloning of pCAG-Myc-DEST-IRES-Puro | CTTCTGATAGGCAGCCTGCACCTGAGGAGTGCGGCCGCtcaggcaccgggcttgcg | Not I |
| Hifi cloning of pCAG-Myc-DEST-IRES-Puro-T2A-LifeActEGFP | CTTCTGATAGGCAGCCTGCACCTGAGGAGTGCGGCCGttacttgtacagctcgtcc |  |
| to clone STOp and AseI site into BamHi and EcoRI of pENTR-3C | Phos-GATCCTAGATTAATG |  |
| to clone STOp and AseI site into BamHi and EcoRI of pENTR-3C | Phos-AATTCATTAATCTAG |  |
|  |  |  |
|  |  |  |
| Hifi cloning of ARPC1BmVenus-P2A-ARPC3-Turq2/TEAL | ACTGGATCCGGTACCGAATTCgccaccatggcctaccacag |  |
| Hifi cloning of ARPC1BmVenus-P2A-ARPC3-Turq2/TEAL | cttccGAATTCcttgtacagctcgtccatgcc |  |
| Hifi cloning of ARPC1BmVenus-P2A-ARPC3-Turq2/TEAL | gtacaagGAATTCggaagcggagctactaacttc |  |
| Hifi cloning of ARPC1BmVenus-P2A-ARPC3-Turq2/TEAL | ccggcatgctaggtccagggttctcc |  |
| Hifi cloning of ARPC1BmVenus-P2A-ARPC3-Turq2/TEAL | ggacctagcatgccggcttaccactcttc |  |
| Hifi cloning of ARPC1BmVenus-P2A-ARPC3-Turq2/TEAL | TGTACAAGAAAGCTGGGTCTAGAttacttgtacagctcgtccatg |  |
| for HIFI cloning of ARPC1B-mVenus-P2A-ARPC3-TEAL/mTurq2 | ACTGGATCCGGTACCGAATTCgccaccatggcctac |  |
| for HIFI cloning of ARPC1B-mVenus-P2A-ARPC3-TEAL/mTurq2 | gagtggtaagccggcatgctaggtccagggttctcc |  |
| for HIFI cloning of ARPC1B-mVenus-P2A-ARPC3-TEAL/mTurq2 | gaaccctggacctagcatgccggcttaccactcttc |  |
| for HIFI cloning of ARPC1B-mVenus-P2A-ARPC3-TEAL/mTurq2 | AAGCTGGGTCTAGATATCTCGAGttacttgtacagctcgtccatg |  |
|  |  |  |
| HiFi cloning of DEST for pCDNA3.1-DEST-mScarlet-I or mEGFP | ACGACTCACTATAGGGAGACCCAAGCTGGCTAGCACAAGTTTGTACAAAAAAGCTGAAC |  |
| HiFi cloning of DEST for pCDNA3.1-DEST-mScarlet-I or mEGFP | cctcgcccttgctcaccatgACCACTTTGTACAAGAAAGCTG |  |
| HiFi cloning of Scarlet-I or mEGFP for pCDNA3.1-DEST-mScarlet-I or mEGFP | GCTTTCTTGTACAAAGTGGTcatggtgagcaagggcgagg |  |
| HiFi cloning of Scarlet-I or mEGFP for pCDNA3.1-DEST-mScarlet-I or mEGFP | CCACCACACTGGACTAGTGGATCCGAGCTCGGTACCttacttgtacagctcgtccatg |  |
|  |  |  |
| Hifi cloning of pCAG-Myc-DEST-IRES-Blasticidin | TGAGACAAAGGCTTGGCCATggtattatcgtgtttttcaaagg |  |
| Hifi cloning of pCAG-Myc-DEST-IRES-Blasticidin | ttgaaaaacacgataataccATGGCCAAGCCTTTGTCTCAAG |  |
| Hifi cloning of pCAG-Myc-DEST-IRES-Blasticidin | CTTCTGATAGGCAGCCTGCACCTGAGGAGTGCGGCCGCTTAGCCCTCCCACACATAACC |  |
|  |  |  |
