# Supplementary material for: Nance-Horan Syndrome-like 1 protein negatively regulates Scar/WAVE-Arp2/3 activity and inhibits lamellipodia stability and cell migration
Source: Nat Commun. 2021 Sep 28;12:5687. doi: 10.1038/s41467-021-25916-6 (PMC8478917; doi:10.1038/s41467-021-25916-6)
Supplement: Supplementary file 15 — Supplementary Software 1 [file 41467_2021_25916_MOESM15_ESM.zip › Law et al NCOMMS-18-26320A-Z custom software /20190211-Analysis of length distribution of lamellipodia.docx]

**Analysis of length distribution of lamellipodia**

**1. Generate TIFF stack with protrusions labelled as blue line and retractions as red lines using MATLAB software.**

- Open MATLAB R2015a: open Matlab Script: “displayAllProtrusionsMK.m” (we are using MATLAB R2015a).
- Select folder with the files 'protrusion%d_vectors.mat' and 'Image_%d_Airyscan Processing.tif' (% is a variable number) obtained from the Danuser Windowing Matlab script.
- For a movie of 119 frames the script takes 7 minutes on a late 2015 iMac with 3.2 GHz Intel Core i5 and 8GB RAM.
- The resulting TIFF stack movie is saved as 'Movie_ID%d.tif' into the same folder

**2. Split TIFF stack in software Fiji into RGB channels.**

- Open TIFF stack in Fiji.
- Under “Image” pull down menue select “split channel”.
- Save blue, red and green channels as TIFF stacks.

**3. Use Metamorph software to measure fiber length of all protruding or retracting lamellipodia edges from all frames of each movie.**

- Open blue or red TIFF stack from 2.
- Under “Measure” pull down menue, select “threshold image inclusive”, then binarize (1 bit).
- Under “Measure” pull down menue, select “calibrate”, calibrate pixel size to 0.12um x 0.12um.
- Save as TIFF stack.
- Under “Measure” pull down menue, select “morphometric analysis”, select “measuring: Fiber length”; Display objects; Configure Log: select only “fiber length”.
- Under “Measure” pull down menue, select “open data log”, “open object log” then select “DDE” and “new sheet or column” (Row move absolute, column move relative).
- Use attached Journal “Matthias-Quant-lamellipodiaPersistence”: Under “Journal” pull down menue, select “journal” “Matthias-Quant-lamellipodiaPersistence” then select “loop for all planes”.
- Under “Measure” pull down menue, select “Logs” – close object log.
- The data for fiber length of all protruding or retracting lamellipodia edges from all frames of each movie is now in an excel spread sheet which can be statistically analysed using for example Graphpad PRISM.
