# Supplementary material for: Nance-Horan Syndrome-like 1 protein negatively regulates Scar/WAVE-Arp2/3 activity and inhibits lamellipodia stability and cell migration
Source: Nat Commun. 2021 Sep 28;12:5687. doi: 10.1038/s41467-021-25916-6 (PMC8478917; doi:10.1038/s41467-021-25916-6)
Supplement: Supplementary file 15 — Supplementary Software 1 [file 41467_2021_25916_MOESM15_ESM.zip › Law et al NCOMMS-18-26320A-Z custom software /20190211-Quantification of lamellipodia protrusion speed.docx]

**Quantification of lamellipodia protrusion speed**

**1. Convert Tiff file from Danuser Matlab Segmentation script into a TIFF file that Fiji can read (required for cell tracking using Fiji plugin).**

- Open MATLAB R2016b: open Matlab Script: “tifs_that_fiji_can_use.m”
- Change the name of 'refined_mask_4-n1-Image 026_Airyscan Processing_c1_*.tif' to the name of your movie that you want to analyse.
- Change the name of 'MK_segmented_26' to the name of the tiff result file, which will be saved into the same folder as the orginal file.
- Run Matlab Script: “tifs_that_fiji_can_use.m”
- Select folder containing refined thresholding masks from the segmentation package of the Danuser Windowing script: For example 'refined_mask_4-n1-Image 026_Airyscan Processing_c1_*.tif'
- The converted tiff movie will be saved into the same folder as the original file.

**2. Track cells for protrusion analysis to identify direction of migration.**

- Open converted TIFF stack in Fiji.
- Under “Edit” pull down menu, select “Invert”, select “yes all images”
- Under “Image” pull down menu, select “properties”, change calibration to
  - Channel 1
  - Slices (z) 1
  - Frames (t) (number of frames of movie)
  - Unit length micrometer
  - pixel width 0.12um (or whatever it is)
  - pixel height 0.12um (or whatever it is)
  - pixel length 0.12um (or whatever it is)
  - Frame interval 5 sec (or whatever it is)
- Under “File” pull down menu, select “save”, select “replace”
- Install the Fiji plugin “Mtrack2”
- Under “Plugin” pull down menu, select “Mtrack2” and then select save results file
- Open Text file in excel and copy coordinates from cell tracking and paste into new spreadsheet and save as “trackresults%d.csv” file (% is a variable number).

**3. Use MATLAB to compute the speed of the all protrusions around the entire circumference of the cell, of all protrusions in the largest lamellipodial protrusion, or of all protrusions within a cone of a defined angle in the direction of migration.**

- Open MATLAB R2016b: open Matlab Script: “main.m”
- **For analysis of all protrusions within a cone of a defined angle in the direction of migration:** Define the desired angle for analysis of protrusion dynamics within a cone of cos written in the MATLAB script as “theta = 60;” for Cos theta 60
- Define the pixel size in micrometer (for example for a pixel size of 0.12 um x 0.12 um) write “scale = 0.12;”
- Run Matlab Script: “main.m” and select folder containing the files 'protrusion%d_vectors.mat' and 'trackresults%d.csv'

(% is a variable number) obtained from the Danuser Windowing Matlab script and the tracking from point 2 above. (example files can be found in the folder “20190201-Examples for-protrusion analysis”.

- An option window opens with the options to compute the speed of **“All Protrusions”** (computes the average speed of all protrusions and retractions around the entire circumference of the cell) or **“Largest Area”** (computes the average speed of the protrusions and retractions of the largest protrusive area of the cell) of **“Cone”** (computes the average speed of the protrusions and retractions of a cone with a defined angle towards the directions of migration of the cell).
- Extract the results from the variable “data”: The first column is the magnitude of the vector and the second the number of vectors = pixels along the protrusive edge.
- In Excel from the magnitude of the vector you can compute the speed of the protrusion at this pixel on the edge by multiplying the magnitude with *0.12 (the pixel size in micrometer) *12.
- In Excel from the number of vectors = pixels along the protrusive edge you can compute the width of the protrusion in micrometer by multiplying the number of vectors = pixels with *0.12 (the pixel size in micrometer).
- The data for protrusion speed and width of lamellipodia edges from all frames of each movie is now in an excel spread sheet which can be statistically analysed using for example Graphpad PRISM.
