# Supplementary material for: Nance-Horan Syndrome-like 1 protein negatively regulates Scar/WAVE-Arp2/3 activity and inhibits lamellipodia stability and cell migration
Source: Nat Commun. 2021 Sep 28;12:5687. doi: 10.1038/s41467-021-25916-6 (PMC8478917; doi:10.1038/s41467-021-25916-6)
Supplement: Supplementary file 16 — Reporting Summary [file 41467_2021_25916_MOESM16_ESM.pdf]

nature portfolio

Corresponding author(s): Matthias Krause
Last updated by author(s): Jul 25, 2021

Reporting Summary

Nature Portfolio wishes to improve the reproducibility of the work that we publish. This form provides structure for consistency and transparency in reporting. For further information on Nature Portfolio policies, see our Editorial Policies and the Editorial Policy Checklist.

Statistics

For all statistical analyses, confirm that the following items are present in the figure legend, table legend, main text, or Methods section.

n/a	Confirmed	
☐	☒	The exact sample size (n) for each experimental group/condition, given as a discrete number and unit of measurement
☐	☒	A statement on whether measurements were taken from distinct samples or whether the same sample was measured repeatedly
☐	☒	The statistical test(s) used AND whether they are one- or two-sided<br>*Only common tests should be described solely by name; describe more complex techniques in the Methods section.*
☒	☐	A description of all covariates tested
☐	☒	A description of any assumptions or corrections, such as tests of normality and adjustment for multiple comparisons
☐	☒	A full description of the statistical parameters including central tendency (e.g. means) or other basic estimates (e.g. regression coefficient) AND variation (e.g. standard deviation) or associated estimates of uncertainty (e.g. confidence intervals)
☐	☒	For null hypothesis testing, the test statistic (e.g. F, t, r) with confidence intervals, effect sizes, degrees of freedom and P value noted<br>*Give P values as exact values whenever suitable.*
☒	☐	For Bayesian analysis, information on the choice of priors and Markov chain Monte Carlo settings
☒	☐	For hierarchical and complex designs, identification of the appropriate level for tests and full reporting of outcomes
☐	☒	Estimates of effect sizes (e.g. Cohen's d, Pearson's r), indicating how they were calculated

Our web collection on statistics for biologists contains articles on many of the points above.

Software and code

Policy information about availability of computer code

Data collection	FLIM image acquisition: custom build FLIM microscope controlled by LabView 2018 (National Instruments, Austin, TX).<br>Western Blot Image acquisition: Bio-Rad Imager and ImageLab software version 4.1.<br>Immunofluorescence and live imaging acquisition: IX 81 microscope (Olympus) controlled by Metamorph version 6.1 or a Zeiss LSM880 driven by Zen Black Edition software

Data analysis	FLIM images analysis: in-house exponential fitting algorithm (TRI2 software) written in LabWindows/CVI 2017 (National Instruments, Austin, TX). The TRI2 software for FLIM analysis can be downloaded here: https://flimlib.github.io<br>Actin retrograde flow analysis by Particle Image Velocimitry (PIV): The PIV Matlab script can be downloaded here: https://github.com/stemarcotti/PIV.<br>Immunofluorescence and live imaging postprocessing: Zen Black Edition software; Metamorph offline Version 6.1; Fiji; Adobe Photoshop CS6 and Adobe Photoshop Creative Cloud. Cell profiler analysis of F-actin content in HEK293 cells was analysed using Cellprofiler 2.0 software (http://www.cellprofiler.org, Broad Institute).<br>Cell tracking: Manual Tracking and MTrack2 Plugin (Fiji); Mathematica for analysis using the Chemotaxis Analysis Notebook v1.6β by G. Dunn,King's College London, England, UK (published in ref 6).<br>Analysis of LifeAct-EGFP movies: automatically segmented and protrusion vectors at each pixel along the cell edge calculated using a MATLAB script kindly provided by Gaudenz Danuser (UT Southwestern, USA) available at https://github.com/DanuserLab/Windowing-Protrusion. Custom written MATLAB scripts and Metamorph journals for post analysis of this raw data were used to calculate protrusion speed, the longest uninterrupted lamellipodium, and the length distribution of protrusions from all frames of the movie (provided as Supplementary software 1). Fiji 1.53c and Excel 2016 and 2018 for Mac was used to quantify the LifeAct-EGFP intensity in the first frame of each movie using Fiji.<br>Statistical analysis was performed in Prism v7, v8, v9 (GraphPAD Software)

For manuscripts utilizing custom algorithms or software that are central to the research but not yet described in published literature, software must be made available to editors and reviewers. We strongly encourage code deposition in a community repository (e.g. GitHub). See the Nature Portfolio guidelines for submitting code & software for further information.

Data

Policy information about availability of data

All manuscripts must include a data availability statement. This statement should provide the following information, where applicable:

- Accession codes, unique identifiers, or web links for publicly available datasets
- A description of any restrictions on data availability
- For clinical datasets or third party data, please ensure that the statement adheres to our policy

The imaging datasets generated are available from the corresponding author on reasonable request. All quantifications and full western blots from this study are provided in the Source Data file.

Field-specific reporting

Please select the one below that is the best fit for your research. If you are not sure, read the appropriate sections before making your selection.

☒ Life sciences ☐ Behavioural & social sciences ☐ Ecological, evolutionary & environmental sciences

For a reference copy of the document with all sections, see nature.com/documents/nr-reporting-summary-flat.pdf

Life sciences study design

All studies must disclose on these points even when the disclosure is negative.

Sample size	No Sample size calculations were performed. Sample sizes were chosen following examples of similar experiments done previously in the literature (see for example: Law et al., 2013 JCB DOI: 10.1083/jcb.201304051; Kage et al., 2017 Nat Comm DOI: 10.1038/ncomms14832)
Data exclusions	According to the following pre-established quality criteria and equally applied to all experimental groups the following exclusions were made: In the FLIM experiments for Fig 7 c, f; Suppl Fig 13, 14 and Arp2/3 and in the F-actin intensity measurements for Fig 8 d, e; Suppl Fig 15a-c only cells which displayed a lamellipodium were included. Furthermore, in these experiments also cells which overlapped with other cells or cells with the majority of the lamellipodium displaying ruffling were excluded. In addition, for the FLIM experiments where edge measurements repeatedly (3-5 different sites) returned less than 1000 photons, these cells were also excluded due to low signal-to-noise ratios and low confidence in fitting the decay curves using the Levenberg-Marquart algorithm.
Replication	The data was replicated the number of times as stated in the figure legends.
Randomization	samples were randomly allocated to experimental groups.
Blinding	investigators were blinded during data acquisition and/or analysis as indicated in the manuscript.

Reporting for specific materials, systems and methods

We require information from authors about some types of materials, experimental systems and methods used in many studies. Here, indicate whether each material, system or method listed is relevant to your study. If you are not sure if a list item applies to your research, read the appropriate section before selecting a response.

Materials & experimental systems

n/a	Involved in the study
☐	☒ Antibodies
☐	☒ Eukaryotic cell lines
☒	☐ Palaeontology and archaeology
☒	☐ Animals and other organisms
☒	☐ Human research participants
☒	☐ Clinical data
☒	☐ Dual use research of concern

Methods

n/a	Involved in the study
☒	☐ ChIP-seq
☒	☐ Flow cytometry
☒	☐ MRI-based neuroimaging

Antibodies

Antibodies used

Monoclonal mouse anti-NHSL1 antibody: this study; Polyclonal rabbit anti-NHSL1 antiserum #4457 (custom produced by Eurogentec), this study.
Commercial primary antibodies: EGFP ((11814460001, Roche)), Myc (M5546, 9E10, Sigma), MBP (E8032S, New England Biolabs), Abi1 (MBL, clone 1B9, D147-3), Scar/WAVE1 (BD 612276), Scar/WAVE2 rabbit mAb (D2C8, CST 3659), ARPC2 (07-227-I-100UG, Millipore).
Secondary antibodies: HRP-goat anti-rabbit (P044801, Agilent-Dako), -goat anti-mouse (P044701, Agilent-Dako).

Validation

Validation for custom made antibodies (this study): The NHSL1 monoclonal antibody was subcloned twice (clone C286F5E1; IgG1). The specificity of both antibodies was tested by western blot on cell lysates of the mouse cell line B16-F1 and the human cell line MCF10A and on cell lysates of murine NHSL1 B16-F1 CRISPR KO cells. Please see figures: 1, 2, Suppl Fig 1, 3.
Validation for commercial antibodies: (for validation data see the vendors web sites - see below)
EGFP ((11814460001, Roche)) see https://www.sigmaaldrich.com/GB/en/product/roche/11814460001
Myc (M5546, 9E10, Sigma), see https://www.sigmaaldrich.com/GB/en/product/sigma/m5546?context=product
MBP (E8032S, New England Biolabs), see https://www.neb.uk.com/products/neb-catalogue/protein-analysis,-exp-purification/anti-mbp-monoclonal-antibody
Abi1 (MBL, clone 1B9, D147-3), see https://www.mblintl.com/products/d147-3/
Scar/WAVE1 (BD 612276), no longer commercially available
Scar/WAVE2 rabbit mAb (D2C8, CST 3659), see https://www.cellsignal.co.uk/products/primary-antibodies/wave-2-d2c8-xp-rabbit-mab/3659
ARPC2 (07-227-I-100UG, Millipore), see https://www.merckmillipore.com/GB/en/product/Anti-p34-Arc-ARPC2,MM_NF-07-227-I-100UG
Secondary antibodies:
HRP-goat anti-rabbit (P044801, Agilent-Dako), see https://www.agilent.com/en/product/immunohistochemistry/antibodies-controls/secondary-antibodies/goat-anti-rabbit-immunoglobulins-hrp-(affinity-isolated)-153244
HRP-goat anti-mouse (P044701, Agilent-Dako). see https://www.agilent.com/en/product/immunohistochemistry/antibodies-controls/secondary-antibodies/goat-anti-mouse-immunoglobulins-hrp-(affinity-isolated)-153239

Eukaryotic cell lines

Policy information about cell lines

Cell line source(s)

HEK 293FT cells (Thermo Fisher Scientific R70007) and B16-F1 mouse melanoma cells (ATCC CRL-6323) ; MCF10A cells (ATCC CRL- 10317)

Authentication

none of theses cell lines were independently authenticated

Mycoplasma contamination

All cell lines tested negatively for mycoplasma routinely on a monthly basis.

Commonly misidentified lines
(See ICLAC register)

Cell lines used in this study are not on the list of commonly misidentified cell lines.
